# Multitier regulation of the *E. coli* extreme acid stress response by CsrA

**DOI:** 10.1128/jb.00354-23

**Published:** 2024-02-06

**Authors:** Mark G. Gorelik, Helen Yakhnin, Archana Pannuri, Alyssa C. Walker, Christine Pourciau, Daniel Czyz, Tony Romeo, Paul Babitzke

**Affiliations:** 1 Department of Microbiology and Cell Science, Institute of Food and Agricultural Sciences, University of Florida, Gainesville, Florida, USA; 2 Department of Biochemistry and Molecular Biology, Center for RNA Molecular Biology, The Pennsylvania State University, University Park, Pennsylvania, USA; NCBI, NLM, National Institutes of Health, Bethesda, Maryland, USA

**Keywords:** CsrA, acid stress, posttranscriptional regulation, translation regulation, protein-RNA interaction

## Abstract

CsrA is an RNA-binding protein that regulates processes critical for growth and survival, including central carbon metabolism, motility, biofilm formation, stress responses, and expression of virulence factors in pathogens. Transcriptomics studies in *Escherichia coli* suggested that CsrA repressed genes involved in surviving extremely acidic conditions. Here, we examine the effects of disrupting CsrA-dependent regulation on the expression of genes and circuitry for acid stress survival and demonstrate CsrA-mediated repression at multiple levels. We show that this repression is critical for managing the trade-off between growth and survival; overexpression of acid stress genes caused by *csrA* disruption enhances survival under extreme acidity but is detrimental for growth under mildly acidic conditions. *In vitro* studies confirmed that CsrA binds specifically to mRNAs of structural and regulatory genes for acid stress survival, causing translational repression. We also found that translation of the top-tier acid stress regulator, *evgA*, is coupled to that of a small leader peptide, *evgL*, which is repressed by CsrA. Unlike dedicated acid stress response genes, *csrA* and its sRNA antagonists, *csrB* and *csrC,* did not exhibit a substantial response to acid shock. Furthermore, disruption of CsrA regulation of acid stress genes impacted host-microbe interactions in *Caenorhabditis elegans*, alleviating GABA deficiencies. This study expands the known regulon of CsrA to genes of the extreme acid stress response of *E. coli* and highlights a new facet of the global role played by CsrA in balancing the opposing physiological demands of stress resistance with the capacity for growth and modulating host interactions.

**IMPORTANCE:**

To colonize/infect the mammalian intestinal tract, bacteria must survive exposure to the extreme acidity of the stomach. *E. coli* does this by expressing proteins that neutralize cytoplasmic acidity and cope with molecular damage caused by low pH. Because of the metabolic cost of these processes, genes for surviving acid stress are tightly regulated. Here, we show that CsrA negatively regulates the cascade of expression responsible for the acid stress response. Increased expression of acid response genes due to *csrA* disruption improved survival at extremely low pH but inhibited growth under mildly acidic conditions. Our findings define a new layer of regulation in the acid stress response of *E. coli* and a novel physiological function for CsrA.

## INTRODUCTION

Bacteria have sophisticated regulatory systems that detect and respond to environmental changes. An important example is the carbon storage regulatory (Csr) system, which is present in *E. coli* and other species. The Csr system plays critical regulatory roles in biofilm formation, central carbon metabolism, stress response systems, motility, quorum sensing, and virulence factor expression in pathogens (1–7). The central component of the Csr system is CsrA, a homodimeric protein that recognizes and binds to specific RNA sequences (1, 7). CsrA generally represses stress responses and systems associated with the stationary phase of growth while activating the expression of genes associated with exponential growth (7, 8). CsrA-mediated regulation involves binding to sites containing a critical GGA motif, which is often found in the single-stranded loop of an RNA hairpin (9, 10). These binding sites are typically located in the 5′ leader or early mRNA coding regions. CsrA binding can regulate translation initiation, RNA stability, riboswitch activity, or transcription elongation (5, 7, 11–18).

The expression of *csrA* is tightly regulated, both transcriptionally and posttranscriptionally (19). In addition, CsrA activity is extensively regulated by other components of the Csr system. In *E. coli*, two small RNA (sRNA) antagonists, CsrB and CsrC, contain multiple high-affinity CsrA binding sites that act to sequester CsrA from other regulatory targets (20, 21). *csrB/C* transcription is activated in response to environmental stresses, the accumulation of metabolic end products such as acetate, and quorum sensing (22, 23). CsrB/C levels are also regulated by CsrD *via* RNase E-dependent turnover, which is activated by glucose in *E. coli* (24–27). These findings imply that elevated CsrB/C levels cause decreased CsrA activity under environmental stresses and upon metabolic end product accumulation but increased activity in the presence of preferred carbon sources.

**Fig 1:**
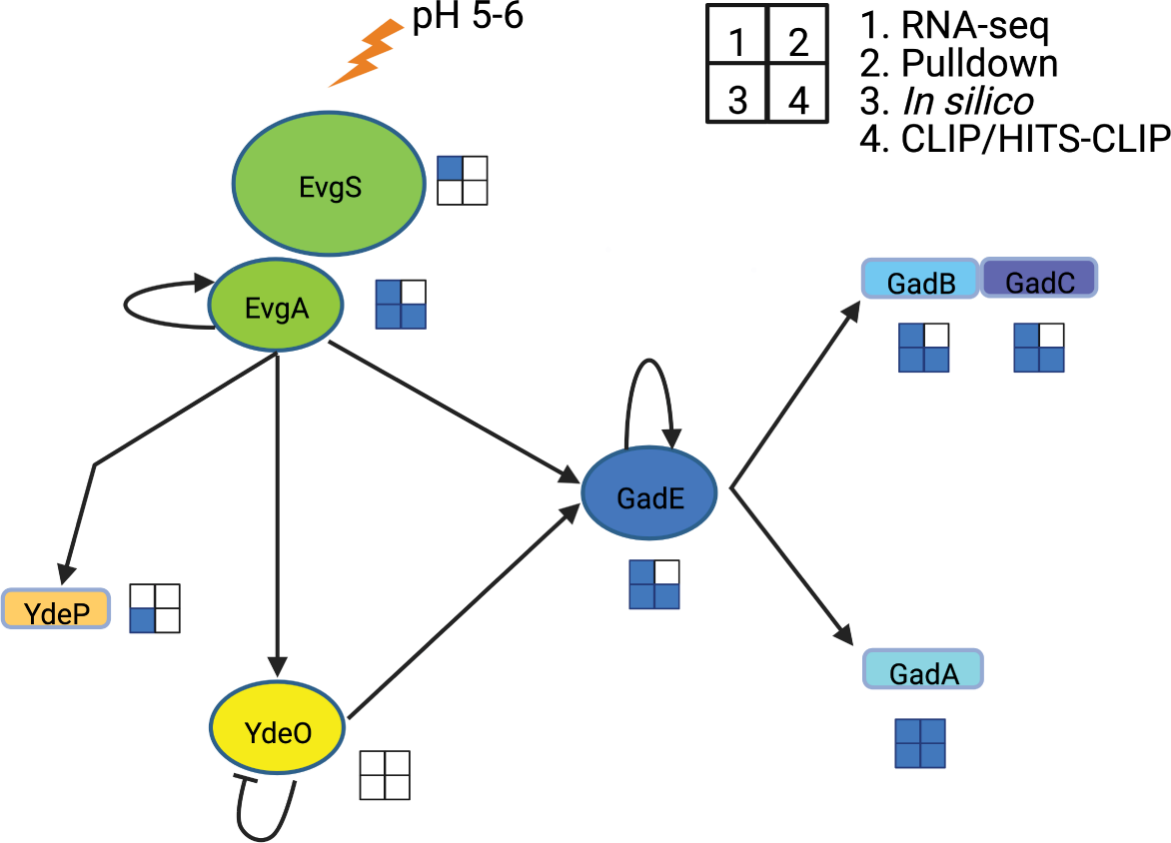
Hypothetical targets of CsrA-dependent regulation in the EvgA-YdeO-GadE circuit. RNA-seq studies include Log_2_ fold changes > 1 (3, 24, 28). Pulldown refers to CsrA-bound RNA targets (29). *In silico* predictions refer to the computational identification of putative CsrA-binding sites (30). CLIP-seq (3) and HITS-CLIP-seq (28) refer to CsrA-binding targets identified by these methods. Blue squares indicate that the gene was identified as a possible regulatory target of CsrA in the respective study.

Recent high-throughput studies identified CsrA as a likely regulator of acid stress resistance genes in *E. coli* (Fig. 1) (3, 9, 28–30). Despite being characterized as a neutrophile, *E. coli* can survive extremely acidic conditions for extended periods of time (31). Tolerance for extremely acidic environments is thought to protect against exposure to gastric acidity (32) and contribute to the low oral infectious dose of certain *E. coli* pathogens (33, 34).

Extremely acidic conditions harm cells by damaging lipids, DNA and RNA, denaturing proteins, and disrupting metabolism (31–33). *E. coli* utilizes several strategies to survive and grow under acidic conditions, including systems that leverage metabolic reactions to neutralize the internal pH (31–33, 35–37). The most critical system for survival in extremely acidic conditions is the glutamate-dependent acid resistance (GDAR) system (31, 38, 39). Three proteins constitute GDAR, including GadA and GadB, which convert glutamate to CO_2_ and γ-aminobutyric acid (GABA), and GadC, which imports glutamate and exports GABA (Fig. 1) (40, 41). The GDAR system is activated by acidic conditions of ~pH 5.5. When the environmental pH drops, GadB and presumably GadA associate with GadC and adopt an active conformation (40, 42, 43). GABA is an important neurotransmitter and has been suggested to be a critical interkingdom signaling molecule, playing roles in the gut-brain axis, in part through organisms carrying the GDAR system (44–46). Another protein critical for acid resistance is YdeP, which can support survival during exponential growth in the absence of exogenous amino acids through an unknown mechanism (Fig. 1) (39, 47–49).

Transcriptional regulation of GDAR and *ydeP* is complex and involves several circuits such as EvgA-YdeO-GadE that activate the expression of *gadA*, *gadBC*, and *ydeP* during the exponential phase (Fig. 1) (39, 50). The EvgA-YdeO-GadE circuit responds to mildly acidic pH when the sensor kinase EvgS phosphorylates the response regulator EvgA (Fig. 1) (51, 52). EvgA in turn activates the transcription of *ydeP* and overexpression of *evgA* leads to increased expression of *gadA* and *gadB* (53). EvgA also activates the transcription of *ydeO* and *gadE*. YdeO is a transcription factor that activates *gadE* transcription, represses its expression, and indirectly represses *ydeP* expression (39, 50, 54). GadE is a transcription factor that is critical for the expression of the GDAR system and for activating its expression. GadE is under complex transcriptional and posttranscriptional regulation involving over a dozen regulators (55–57).

The goal of our study was to investigate CsrA-dependent regulation of the acid stress response in *E. coli*. We demonstrate that CsrA represses acid stress circuitry at multiple levels. Disruption of CsrA causes overexpression of this circuitry, conferring a survival advantage under extremely acidic conditions but a growth defect under mildly acidic conditions. Our findings highlight a new and important role played by CsrA in managing the trade-off between bacterial growth and stress survival.

## RESULTS

### Disruption of *csrA* causes a pH-dependent growth defect

**Fig 2:**
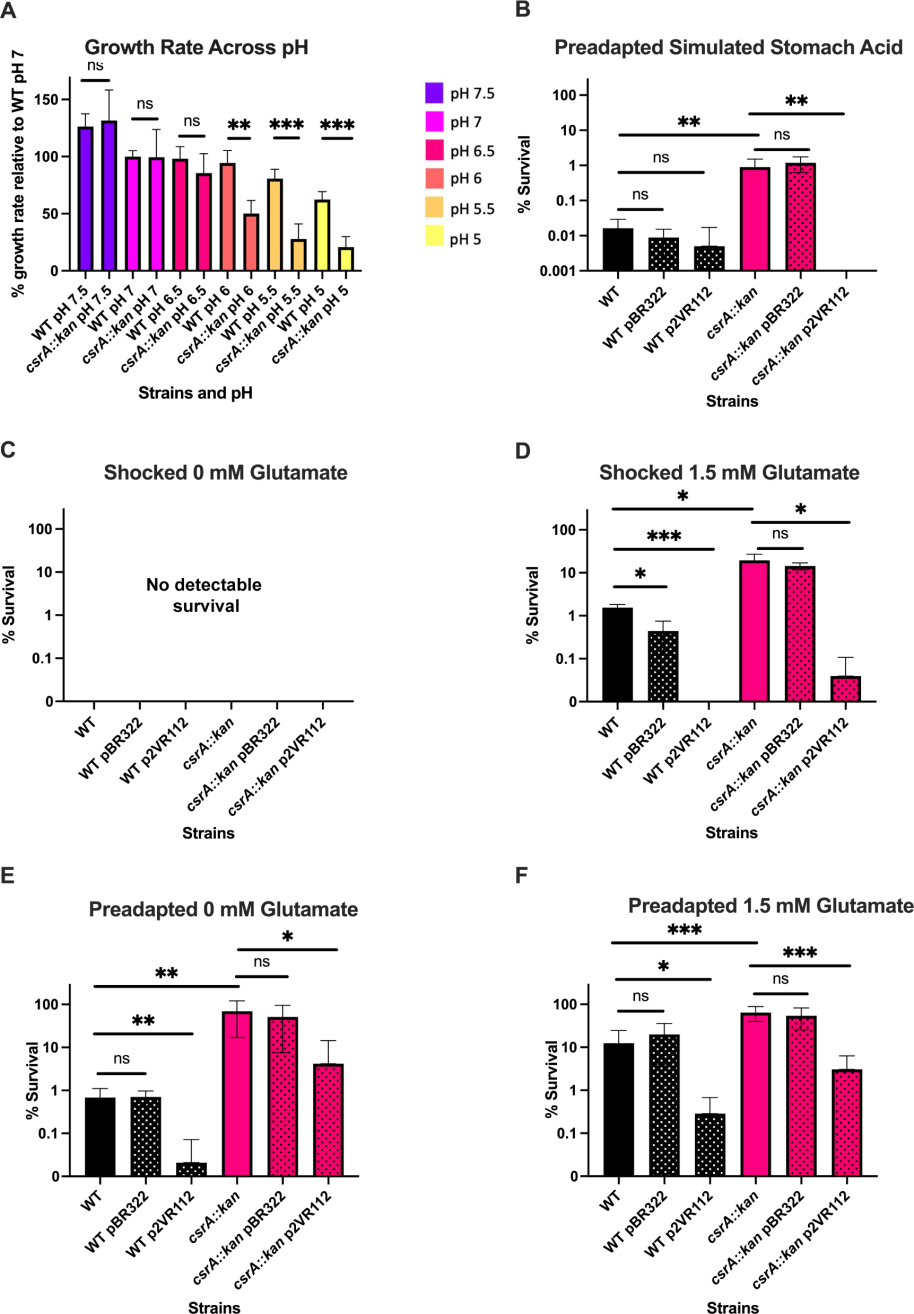
The *csrA* mutant exhibits pH-dependent growth and survival phenotypes. pBR322 is a control plasmid and p2VR112 overexpresses *csrA*. Stippling indicates that the strain carries a plasmid. (**A**) Relative growth rates of *E. coli* MG1655 (WT) and its isogenic *csrA::kan* mutant are shown. Error bars represent the standard deviation (sd) from four independent experiments. (**B**) Cells grown exponentially under mildly acidic conditions and then challenged by simulated stomach acid (pH 1.6) for 10 min. (**C**) Cells grown exponentially under neutral conditions and then challenged by extremely acidic conditions without glutamate (0 mM glutamate) for 2 h. No survival of any strain was seen under these conditions. (**D**) Cells grown exponentially under neutral conditions and then challenged by extremely acidic conditions with 1.5 mM glutamate for 2 h. (**E**) Cells grown exponentially under mildly acidic conditions and then challenged by extremely acidic conditions without glutamate for 2 h. (**F**) Cells grown exponentially under mildly acidic conditions and then challenged by extremely acidic conditions with 1.5 mM glutamate for 2 h. Error bars represent standard deviation (sd) from at least three independent experiments. Statistical significance was determined using unpaired *t*-tests and is denoted as follows: *P < 0.05; **P < 0.01; ***P < 0.001.

Recent studies identified several likely mRNA targets of CsrA-dependent regulation involved in growth and survival in acidic conditions including *evgA* and *gadA* (Fig. 1; Table S1) (3, 9, 28–30). To assess the physiological implications of CsrA-dependent regulation of acid stress systems, wild-type (WT) *E. coli* strain MG1655 and its isogenic *csrA::kan* mutant were grown under different pH conditions ranging from 7.5 to 5 (Fig. 2A; Table S1). At pH 6 or below, the *csrA* mutant displayed a strong growth defect that intensified as the pH decreased further. While the growth rate of the WT strain also decreased under acidic conditions, the effect was substantially weaker. Complementing the *csrA* mutation with a plasmid-borne wild-type *csrA* gene restored WT growth at pH 5.5, confirming the role of CsrA in this phenotype (Fig. S2). These results suggest that CsrA represses genes detrimental for the growth under acidic conditions, activates genes beneficial for growth under acidic conditions, or both. Interestingly, pH 6 is the upper limit of activation for the EvgS sensor kinase and increased activity and expression of the EvgAS TCS are associated with a pH-dependent growth defect, suggesting that CsrA may negatively regulate EvgAS function or downstream regulatory targets of EvgAS (47, 49).

### CsrA regulates survival under extremely acidic conditions

When *E. coli* cells were grown in neutral media (pH 7) and then shocked by exposure to extremely acidic conditions (pH 2.5) in the absence of exogenous glutamate, little to no survival was observed for WT or *csrA*-mutant strains (Fig. 2C). However, when shocked under extremely acidic conditions in the presence of glutamate, mean survival of the *csrA* mutant was ~12 fold higher than WT, suggesting that the GDAR system is more highly expressed or more active in the *csrA* mutant (Fig. 2D; Table S1). Complementation of the *csrA* mutant with plasmid-borne *csrA* reversed this survival phenotype, confirming the role of CsrA in the acid stress response.

When cells were grown in mildly acidic media (pH 5.5), preadapting them before challenge with extreme acidity, the *csrA* mutant exhibited a much higher survival rate than WT in all conditions (Fig. 2B, E, and F). Without added glutamate, the *csrA* mutant survived ~100-fold better than WT, and with added glutamate ~5-fold better than WT (Fig. 2E and F). Complementation of the *csrA* mutation reversed these phenotypes (Fig. 2).

The effect of CsrA on survival also extended to a condition designed to simulate fasted human stomach contents after drinking a glass of water (Biorelevant Media). After a 10-min challenge, the *csrA* mutant survived ~55-fold better than WT. Complementation of *csrA* abolished this survival phenotype (Fig. 2B). These results suggest that CsrA likely impacts survival in stomach acidity during host colonization. Survival under extremely acidic conditions in the absence of external amino acids has been associated with *evgA* and *ydeP* overexpression (49), offering a possible explanation for this phenotype.

### Acid stress phenotypes of the *csrA* mutant depend on the EvgA-YdeO-GadE circuitry

**Fig 3:**
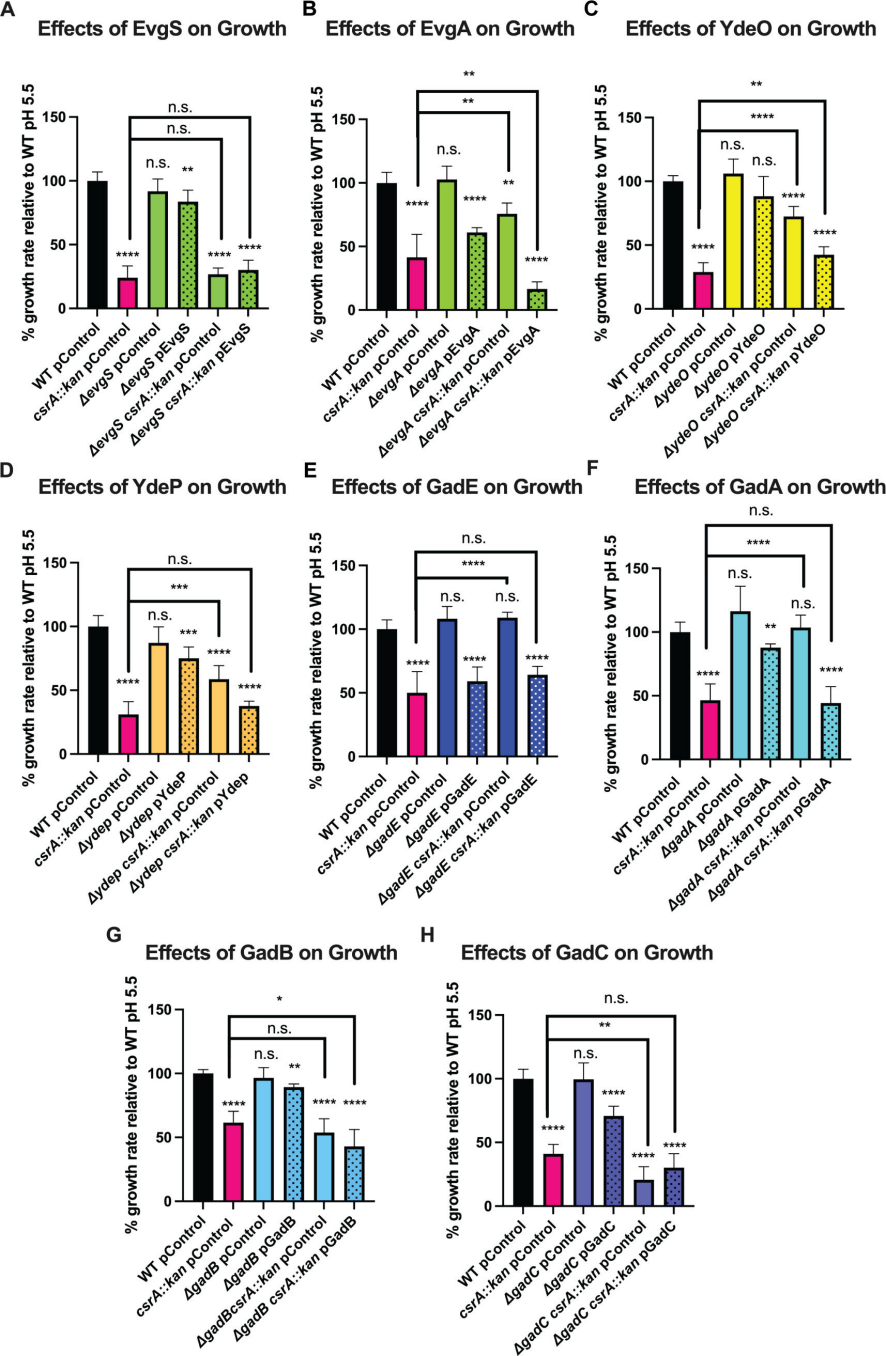
Suppressors of the pH-dependent growth defect of the *csrA* mutant. (**A through H**) Growth rates (μ) of exponential phase *E. coli* strains in mildly acidic (pH 5.5) M9 media normalized to WT. Stippling indicates complemented strains. Gene products of possible suppressors are indicated in the heading of each panel. Error bars represent the standard deviation (sd) from six independent experiments. Statistical significance was determined using an unpaired *t*-test and is denoted as follows: *P < 0.05; **P < 0.01; ***P < 0.001; ****P < 0.0001. The absence of a comparison bar indicates a comparison to WT.

We used epistasis analysis to identify genes responsible for the pH-dependent physiology of the *csrA* mutant (Fig. 1; Table S1). Deletion of *evgS*, *gadB*, or *gadC* did not suppress the growth defect of the *csrA* mutant (Fig. 3A, G, and H). However, deletion of several other genes in the EvgA-YdeO-GadE circuit suppressed the pH-dependent growth defect of the *csrA* mutant grown under mildly acidic conditions, including *evgA, ydeO, ydeP, gadE*, and *gadA* (Fig. 3B through F; Fig. S3). Complementing these genes in mutant strains restored the growth defect of the *csrA* mutant. Previous studies indicated that overexpression of *evgA* and *ydeP* impaired the growth of an otherwise WT strain (49), similar to the growth defective phenotype observed in the *csrA* mutant. Our results suggest that the EvgA-YdeO-GadE circuit participates in the pH-dependent growth defect of the *csrA* mutant and imply that CsrA may regulate the EvgA-YdeO-GadE circuit. Deletion of *evgS*, *gadB*, or *gadC* did not suppress the growth defect of the *csrA* mutant (Fig. 3A, G, and H).

**Fig 4:**
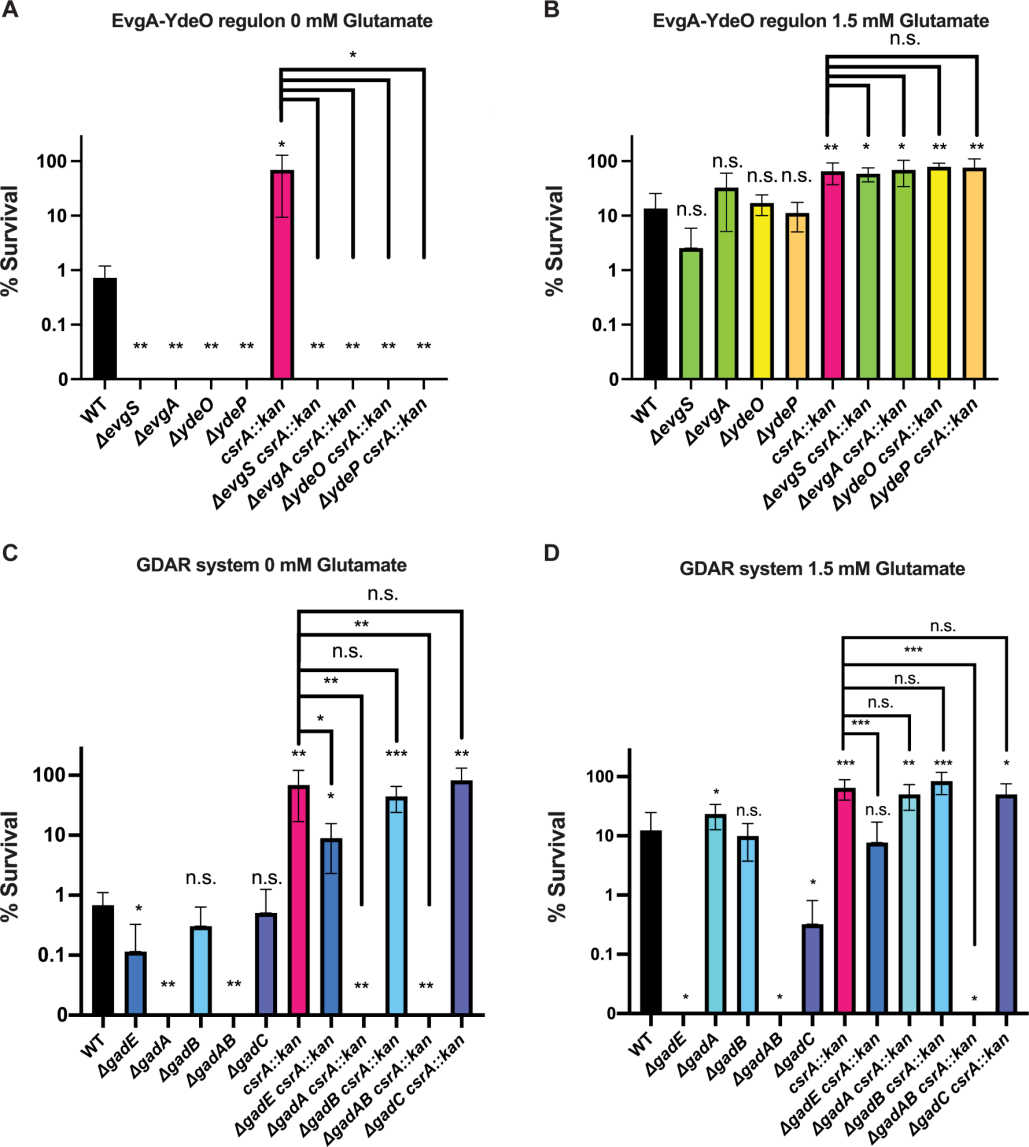
Suppression of the *csrA* mutant survival phenotype in preadapted cells. Exponentially growing cells (OD = 0.5) were preadapted at pH 5.5 and then challenged for 2 h at pH 2.5. (**A**) Knockouts of genes relevant to the EvgA-YdeO regulon were challenged under extremely acidic conditions without glutamate or (**B**) extremely acidic conditions with 1.5 mM glutamate. (**C**) Knockouts of genes relevant to the GDAR system were challenged under extremely acidic conditions without glutamate or (**D**) extremely acidic conditions with 1.5 mM glutamate. Error bars represent the standard deviation (sd) from six independent experiments. Statistical significance was determined using unpaired *t*-tests and is denoted as follows: *P < 0.05; **P < 0.01; ***P < 0.001. The absence of a comparison bar indicates a comparison to WT.

We next tested whether the deletion of genes in the EvgA-YdeO-GadE circuit affected the survival of WT and *csrA* mutant strains that were preadapted by growth under mildly acidic conditions (pH 5.5) and then subjected to extremely acidic conditions (pH 2.5). Deleting *evgS*, *evgA*, *ydeO*, *ydeP*, or *gadA* abolished the survival of WT and *csrA* mutant strains in the absence of exogenous glutamate (Fig. 4A and C). Deleting *gadE* reduced the survival of WT and the *csrA* mutant under extremely acidic conditions with or without added glutamate (Fig. 4C and D). Interestingly, deleting *gadB* did not affect survival with or without glutamate, whereas deleting *gadA* resulted in a survival defect when grown without glutamate but not in its presence (Fig. 4C and D).

Deleting *evgS* in a *csrA* mutant abolished the survival phenotype but it did not alter the growth defect of this strain (Fig. 3A and 4A). This paradox may be due to the influence of two factors: in the absence of EvgS, EvgA can be phosphorylated by acetyl-phosphate and EvgS is thought to be necessary for dephosphorylation of EvgA (58–60). Perhaps, sufficient phosphorylated EvgA accumulates in an *evgS* knockout to cause impaired growth of the *csrA* mutant but not enough to increase survival of the *csrA* mutant.

Glutamate supplementation eliminated survival defects of the *evgS*, *evgA*, *ydeO*, *ydeP*, and *gadA* deletions in WT and *csrA* mutant backgrounds, consistent with similar findings from prior studies (Fig. 4B and D); however, *gadA* was not tested during exponential growth in those studies (39, 48, 49). Deleting either *gadA* or *gadB* when supplemented with glutamate had little to no effect on the survival of the WT or *csrA* mutant strains. However, deleting both *gadA* and *gadB* in the WT and *csrA* mutant backgrounds abolished survival in the presence or absence of glutamate (Fig. 4C and D), highlighting the redundant nature of these genes. Deleting *gadE* impaired the survival of the WT and *csrA* mutant strains when supplemented with glutamate (Fig. 4D), similar to previous observations (61).

### CsrA binds to 5′ leaders of mRNAs in the EvgA-YdeO-GadE circuit

**Fig 5:**
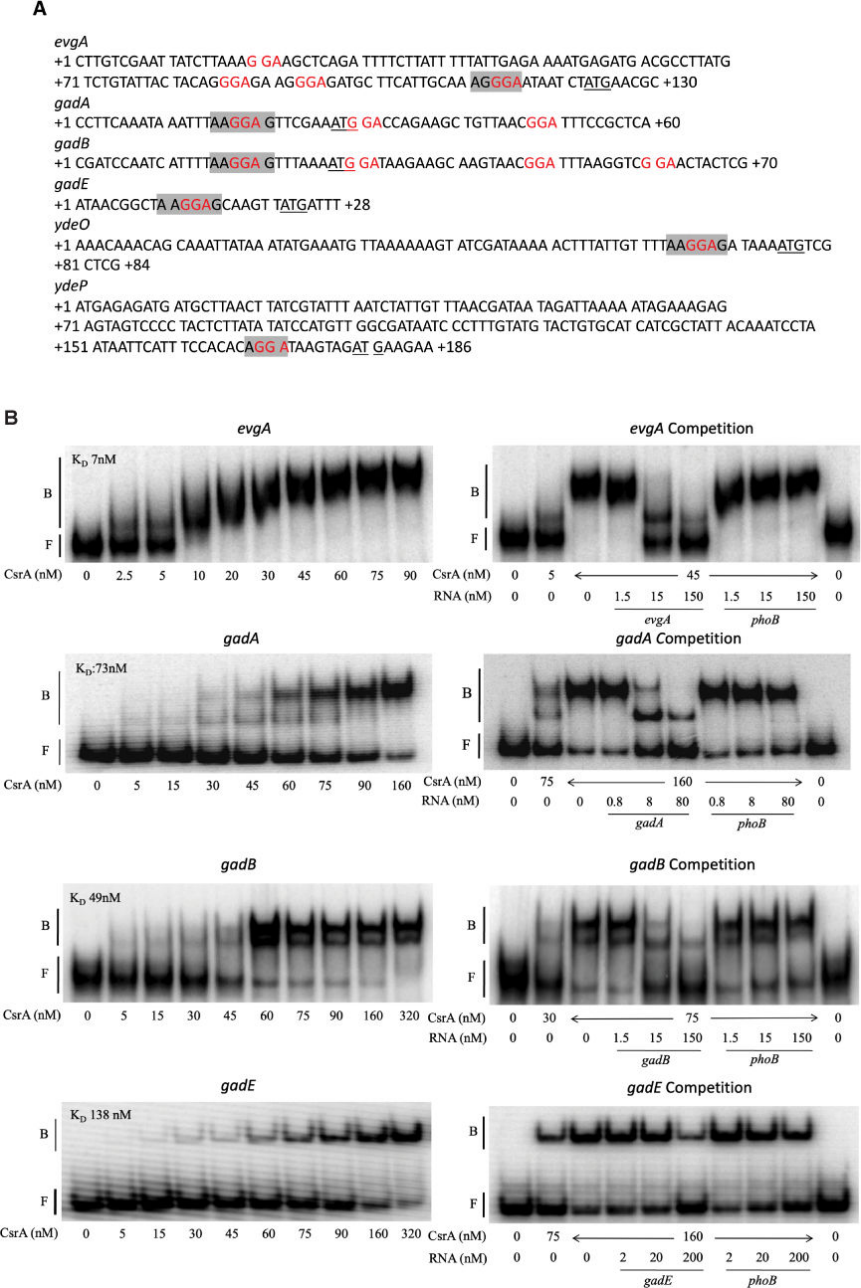
CsrA binding interactions with *evgA*, *gadA*, *gadB*, *gadE*, *ydeO*, and *ydeP* mRNA. (**A**) Sequences of probes used for gel shift assays. GGA motifs that may be components of CsrA binding sites are shown in red, start codons are underlined, and the Shine-Dalgarno (SD) sequence is shaded. (**B**) 5′-end-labeled transcripts were incubated with CsrA at the indicated concentrations. Competition reactions were performed in the presence of unlabeled specific (self) or unlabeled nonspecific (*phoB*) competitor RNAs at the concentrations shown. The positions of free (**F**) and bound (**B**) RNA are marked.

While previous CLIP-Seq and RNA pulldown studies suggested that CsrA binds to *evgA*, *gadE*, *gadA*, and *gadB* mRNAs, these *in vivo* studies did not determine if CsrA binds independently of other factors or assess the binding affinity and specificity (3, 28, 29). Hence, gel shift assays were utilized to explore direct CsrA binding to the 5′ leader of transcripts in the EvgA-YdeO-GadE circuit independently of other factors (Fig. 5A).

Previous studies demonstrated that CsrA-mediated regulation of complex genetic circuitry can occur *via* the top-tier regulator, as is the case for the regulation of motility via *flhDC* (4) or biofilm regulation via *nhaR* (62). EvgA appears to be the top-tier regulator of the acid stress response and is the first gene of the *evgA-evgS* operon. CsrA bound with high affinity to the 5′ leader of the *evgA* transcript (Fig. 5B), with an apparent K_D_ of 7 nM. As the CsrA concentration was increased, two shifted species were observed, suggesting that more than one CsrA dimer can bind to the *evgA* 5′ leader transcript. This RNA contains 4 GGA motifs that might be components of CsrA-binding sites (Fig. 5A). Competition assays with unlabeled specific (*evgA*) and nonspecific (*phoB*) RNA established the specificity of this interaction.

CsrA also bound to the 5′ leaders of *gadA*, *gadB*, *gadE,* and *ydeO* with apparent K_D_ values of 73 nM, 49 nM, 138 nM, and 84 nM, respectively (Fig. 5B). Two distinct shifts were observed for the *gadA* and *gadB* transcripts, suggesting that more than one CsrA dimer may bind to these transcripts. Given that the *gadA* RNA contains 3 GGAs and *gadB* contains 4 GGAs, this explanation is plausible. Competition assays indicated that CsrA binding to both *gadA* and *gadB* RNA was specific (Fig. 5B). On a native gel, the *ydeO* transcript adopted two forms (F1 and F2), and CsrA bound to both forms. Because this transcript produced a single band on a denaturing gel (data not shown), these forms represent alternative RNA conformations. The potential regulatory implications of these two RNA conformations are not known. CsrA did not bind to the 5′ leader of *ydeP,* suggesting that *ydeP* is not a direct target of CsrA-dependent regulation.

Together, these findings reveal that CsrA binds directly to the transcripts of the EvgA-YdeO-GadE circuit in the absence of other factors. Interestingly, CsrA bound most tightly to mRNA of the top-tier regulator EvgA and with weaker affinity to mRNAs of downstream genes within the circuit.

### CsrA regulates *evgA* translation *via* translational coupling to a small leader peptide

**Fig 6:**
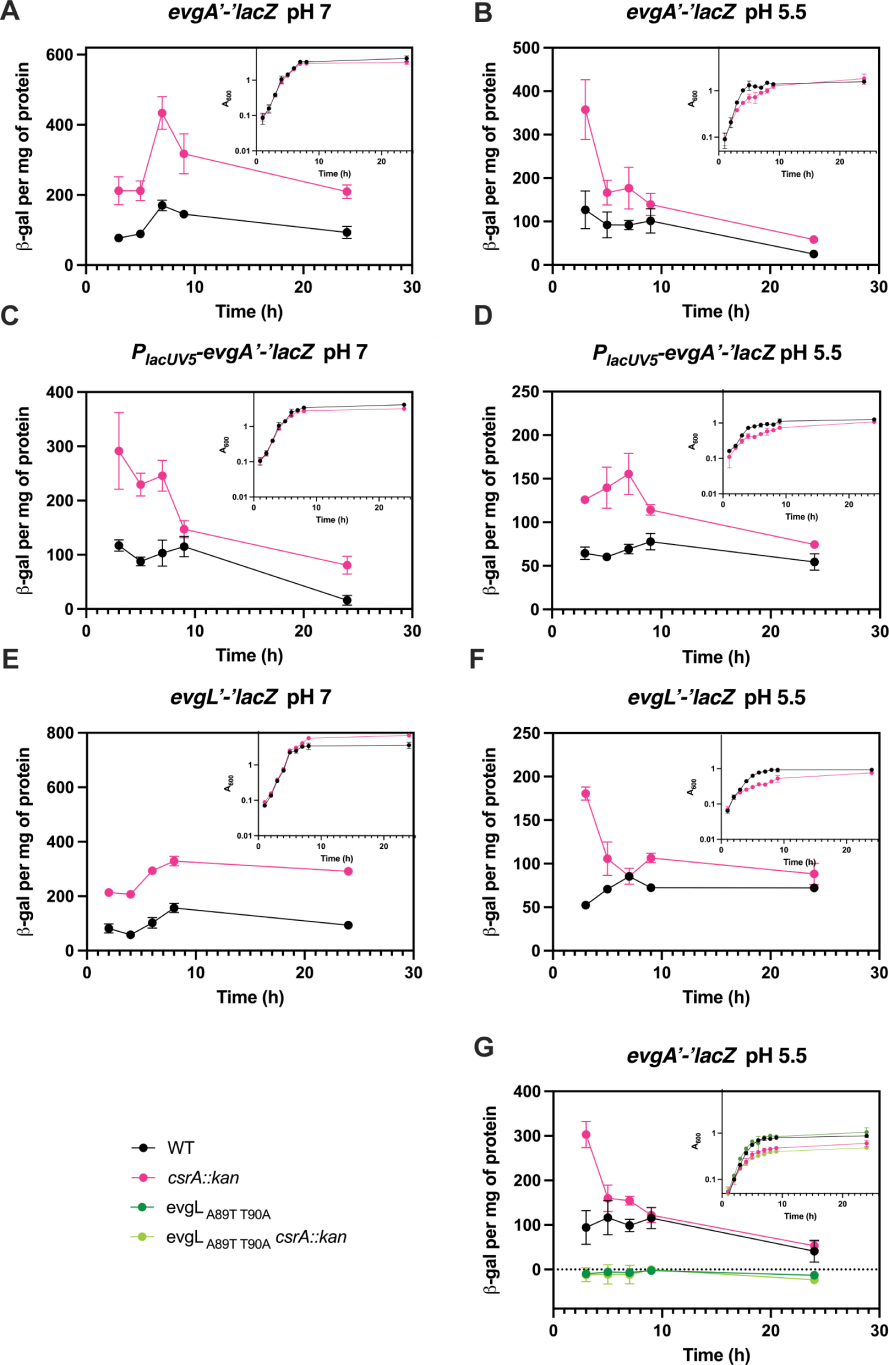
Effects of CsrA on *evgA* expression *in vivo*. Translation fusion expression of *evgA* (**A**) at pH 7 and (**B**) at pH 5.5. Leader fusion expression of *evgA* (**C**) at pH 7 and (**D**) at pH 5.5. Translational fusions of *evgL* (**E**) at pH 7 and (**F**) at pH 5.5. (**G**) Translational fusion expression of *evgA* with the wild type *evgL* start codon mutated to a stop codon (A89T:T90A). Growth curves (OD_600_) are shown in the panel insets. Error bars represent the standard deviation (sd) from three independent experiments.

**Fig 7:**
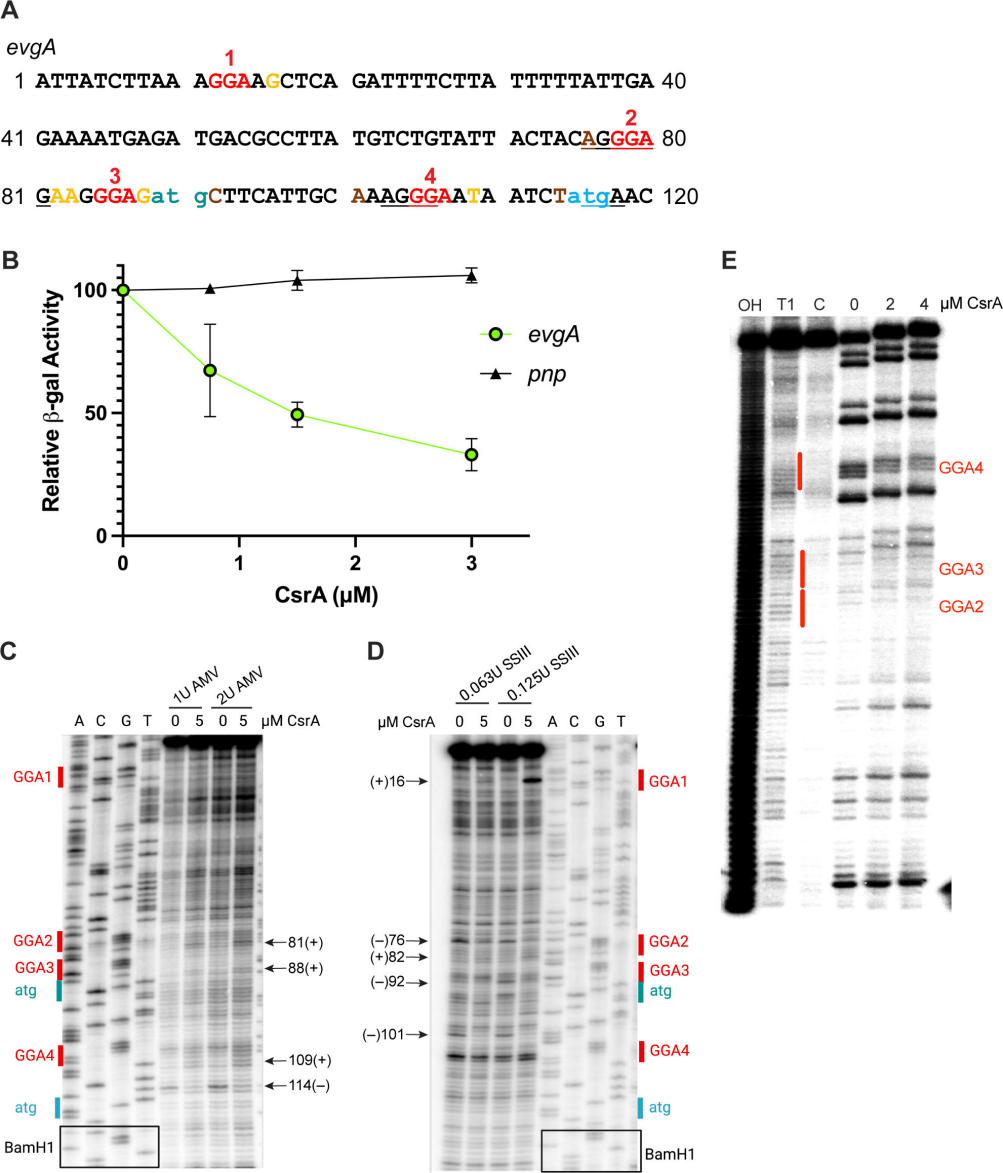
CsrA binding represses translation of *evgA in vitro*. (**A**) *evgA* leader sequence. GGA motifs are shown in red, the start codon of *evgL* in teal, the start codon of *evgA* in blue, and toeprint positions from AMV are in yellow and SSIII is in brown. The *evgL* and *evgA* SD sequences and the *evgL* stop codon are underlined. (**B**) Effects of CsrA on *in vitro* translation of *evgA'-'lacZ* and *pnp'-'lacZ* (control) translational fusions driven by a T7 RNAP promoter. Relative β-galactosidase activity depicts the mean and standard deviation of activity relative to reaction mixtures without CsrA. (**C**) CsrA-dependent toeprints on *evgA* RNA using AMV reverse transcriptase. Nucleotides in red indicate a GGA motif, blue indicates the *evgA* start codon*,* and teal the *evgL* start codon. Arrows (+) indicate bands upon the addition of CsrA and (–) indicate the loss of a band upon the addition of CsrA. (**D**) Toeprinting of the *evgA* transcript using SSIII reverse transcriptase. Color coding and arrows are the same as in (**C**). (**E**) CsrA-*evgA* RNA footprint. 5′-end-labeled *evgA* RNA was treated with RNase T1 ±CsrA, as shown. RNA exposed to partial alkaline hydrolysis (OH), RNase T1 digestion of denatured RNA (**T1**), and untreated control RNA (**C**) are also shown. Red vertical lines correspond to CsrA binding sites that contain the GGA motifs.

Having identified the regulatory circuitry of interest for CsrA effects on acid stress resistance and the genes that CsrA likely regulates directly, we next assessed the effects of CsrA on gene expression using *lacZ* reporter fusions. First, *evgA* expression was examined under neutral and acidic conditions throughout the growth cycle in WT and *csrA* mutant backgrounds. Expression of an *evgA’-‘lacZ* translational fusion was higher in the *csrA* mutant under neutral and acidic conditions (Fig. 6A and B). In addition, a *P_lacUV5_-evgA′-′lacZ* leader fusion was tested in which the *evgA* promoter was replaced with the *lacUV5* promoter. This promoter is unaffected by the *csrA::kan* mutation either directly or indirectly and was used to assess posttranscriptional regulation. The leader fusion was expressed at a higher level in the *csrA* mutant (Fig. 6C and D). Both the translational and leader fusions for *evgA* showed a similar level of higher expression (~2 fold) in the *csrA* mutant, suggesting that the difference of expression of *evgA’-‘lacZ* in the *csrA* mutant is likely a result of disrupted posttranscriptional regulation rather than transcriptional regulation. To determine whether CsrA directly represses *evgA* translation*,* a P_T7_-*evgA’-’lacZ* translational fusion expressed from a T7 promoter was used in the PURExpress system. The decrease in expression caused by increasing concentrations of CsrA indicated that CsrA directly represses *evgA* translation (Fig. 7B).

Recent studies identified a leader peptide, EvgL, in the *evgA* leader mRNA such that the *evgL* stop codon overlaps with the start codon of *evgA,* suggestive of translational coupling (9, 63). We investigated the possibility that *evgA* translation is coupled to that of *evgL* by determining if *evgL* is translated and then by examining the impact of *evgL* translation on *evgA* translation. Hence, we constructed an *evgL’-‘lacZ* translational fusion and found that expression was regulated by CsrA under both neutral and acidic conditions in a pattern similar to the expression of *evgA* (Fig. 6E and F). To assess the effects of *evgL* translation on *evgA* translation, we changed the start codon of *evgL* to a stop codon in the context of an *evgA’-‘lacZ* translational fusion. When *evgL* translation was disrupted by the stop codon, *evgA’-‘lacZ* expression was eliminated, which is indicative of translational coupling (Fig. 6G). Similar CsrA-mediated regulation *via* translational coupling of *iraD* to a short leader peptide was observed previously (64).

RNA footprint and toeprint analyses of CsrA binding suggested that CsrA-mediated regulation of *evgA* expression involves a more complex mechanism. Footprinting with RNase T1 showed CsrA-dependent protection of GGA4, which overlaps the *evgA* SD sequence. Footprints were not observed for the other GGA motifs (Fig. 7E). However, toeprinting assays, which identify the 3′ boundary of a bound protein or stable RNA secondary structure (65), suggested that CsrA has four binding sites in the *evgA* 5′ leader. The use of Avian Myeloblastosis Virus reverse transcriptase (AMV) indicated that CsrA interacts with the two GGAs in the 5′ leader that overlap the *evgL* SD sequence (GGA2) and just upstream of its start codon (GGA3), as well as the GGA overlapping the *evgA* SD sequence (GGA4), while toeprinting with SuperScript III reverse transcriptase (SSIII) suggested that CsrA interacts with GGA1 in the 5′ leader (Fig. 7C and D). These results imply that CsrA regulates the translation of *evgA* directly and *via* translational coupling with *evgL*.

### CsrA regulates the expression of the EvgA-GadE-YdeO circuit at multiple levels

**Fig 8:**
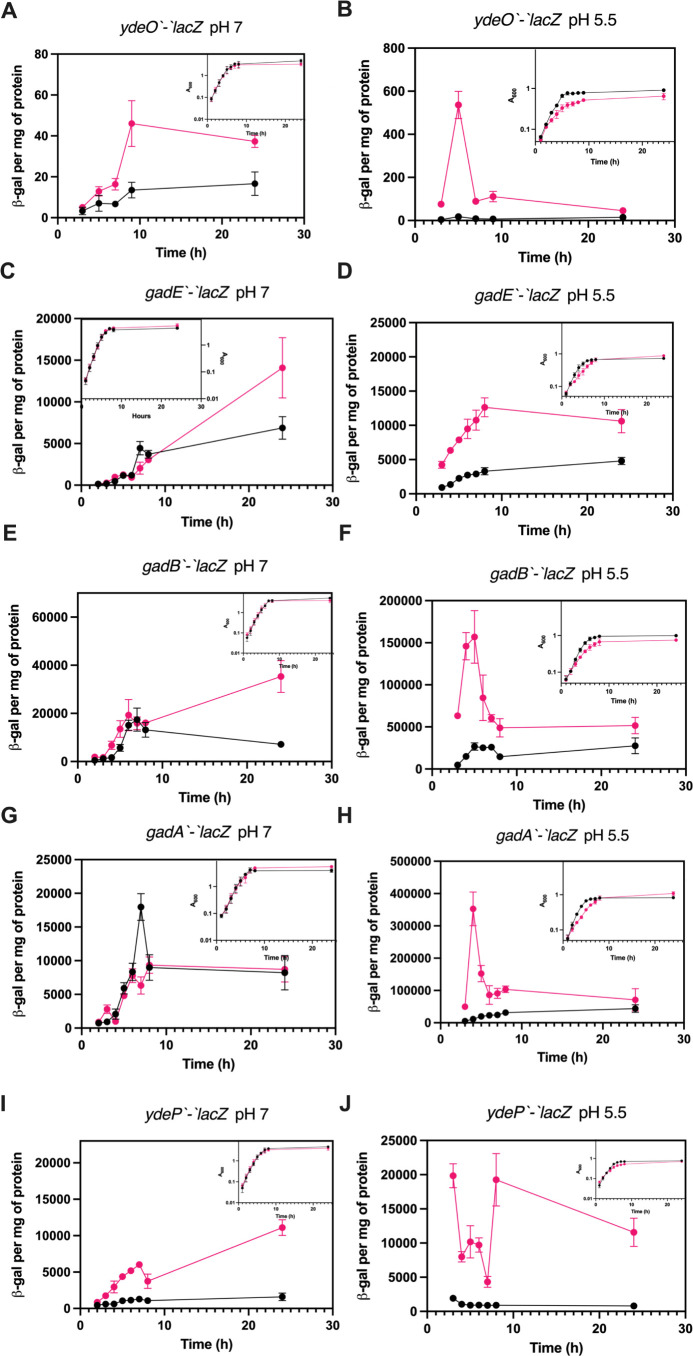
Effects of the *csrA* mutation on expression of *ydeO*, *gadE*, *gadA*, *gadB*, and *ydeP* under neutral and mildly acidic conditions. Expression of *lacZ* translational fusions in WT and *csrA* mutant strains. (**A**) *ydeO* at pH 7, (**B**) *ydeO* at pH 5.5, (**C**) *gadE* at pH 7, (**D**) *gadE* at pH 5.5, (**E**) *gadA* at pH 7, (**F**) *gadA* at pH 5.5, (**G**) *gadB* at pH 7, (**H**) *gadB* at pH 5.5, (**I**) *ydeP* at pH 7, and (**J**) *ydeP* at pH 5.5. Expressions in the WT and *csrA* mutant strains are shown in black and red, respectively. Growth is shown in panel insets. Error bars represent the standard deviation (sd) from three independent experiments.

We next assessed the effects of CsrA on the expression of other acid stress resistance genes using *lacZ* translational fusions for mid-level regulators, *ydeO* and *gadE*, and for genes under the control of EvgA (*ydeP*) or GadE (*gadA* and *gadB*). The *ydeO’-‘lacZ* fusion was expressed at a higher level in the *csrA* mutant strain under neutral (pH 7) and mildly acidic conditions (pH 5.5) (Fig. 8A and B). Expression of the *gadE’-‘lacZ* translational fusion was similar in WT and *csrA* mutant strains under neutral conditions during exponential growth, but the expression in the *csrA* mutant was higher in the stationary phase (Fig. 8C). By contrast, under mildly acidic conditions, *gadE’-‘lacZ* expression was higher in the *csrA* mutant strain throughout growth (Fig. 8D).

Expression of both *gadA’-‘lacZ* and *gadB’-‘lacZ* translational fusions exhibited the effects of CsrA (Fig. 8E through H), the details of which helped to explain their differential effects on physiology, where GadA played an important role in the growth defect of the *csrA* mutant but GadB did not (Fig. 3). Under mildly acidic conditions, both fusions were expressed at higher levels in the *csrA* mutant during the exponential phase of growth. At maximal expression in the *csrA* mutant (~4 h), the *gadA’-‘lacZ* fusion was expressed at a level that was ~2.5-fold higher than the *gadB’-‘lacZ* fusion. In addition, complementing the *gadA* knockout with *gadB* on a plasmid reintroduced the growth defect of the *csrA* mutant, suggesting that the level of expression, and not the particular isozyme, is responsible for the effects on growth (Fig. S4). Under neutral conditions, *gadB’-‘lacZ* expression showed little to no effect of CsrA during the exponential growth but expression was higher in the *csrA* mutant in the stationary phase, while expression of the *gadA’-‘lacZ* fusion was comparable in the WT and *csrA* mutant strains (Fig. 8E and G).

Interestingly, the *ydeP’-‘lacZ* translational fusion was expressed at a higher level throughout growth in the *csrA* mutant strain under both neutral and mildly acidic conditions (Fig. 8I and J). Since CsrA did not bind to the *ydeP* 5′ leader (Fig. 5B), these results imply that CsrA-dependent regulation is mediated indirectly, likely *via* direct effects of CsrA on *evgA* expression.

The translational fusion assays showed that the disruption of CsrA-dependent regulation results in increased expression of genes in the EvgA-YdeO-GadE circuit under acidic conditions, highlighting the importance of CsrA-dependent regulation of the acid stress response. As described above, we observed increased expression of *evgA* in the *csrA* mutant (Fig. 6A and B). While *evgA* did not respond to acidic conditions in the WT background, this was not the case in the *csrA* mutant in which expression increased earlier during growth at ~3 h (Fig. 6A and B). This lack of an *evgA* response to mildly acidic conditions was observed in previous studies using transcriptional fusions, despite the fact that EvgA is reported to be autoregulatory (50, 52, 66). To investigate whether CsrA regulated the circuitry downstream of GadA primarily *via evgA*, we examined the effects of knocking out *evgA* on the expression of a *gadE'-'lacZ* translational fusion. Deletion of *evgA* did not eliminate the effects of CsrA on *gadE* expression (Fig. S5). This result is consistent with the gel shift data indicating that CsrA binds directly to the *gadE* transcript (Fig. 5B) and confirms the multi-tier nature of CsrA-dependent regulation of this circuit. However, *gadE* is subject to several transcription activation and repression mechanisms and is itself positively autoregulatory, which may complicate the interpretation of CsrA effects on *gadE* expression in an *evgA* mutant (50, 67).

### CsrA directly regulates *gadA* and *gadB*

**Fig 9:**
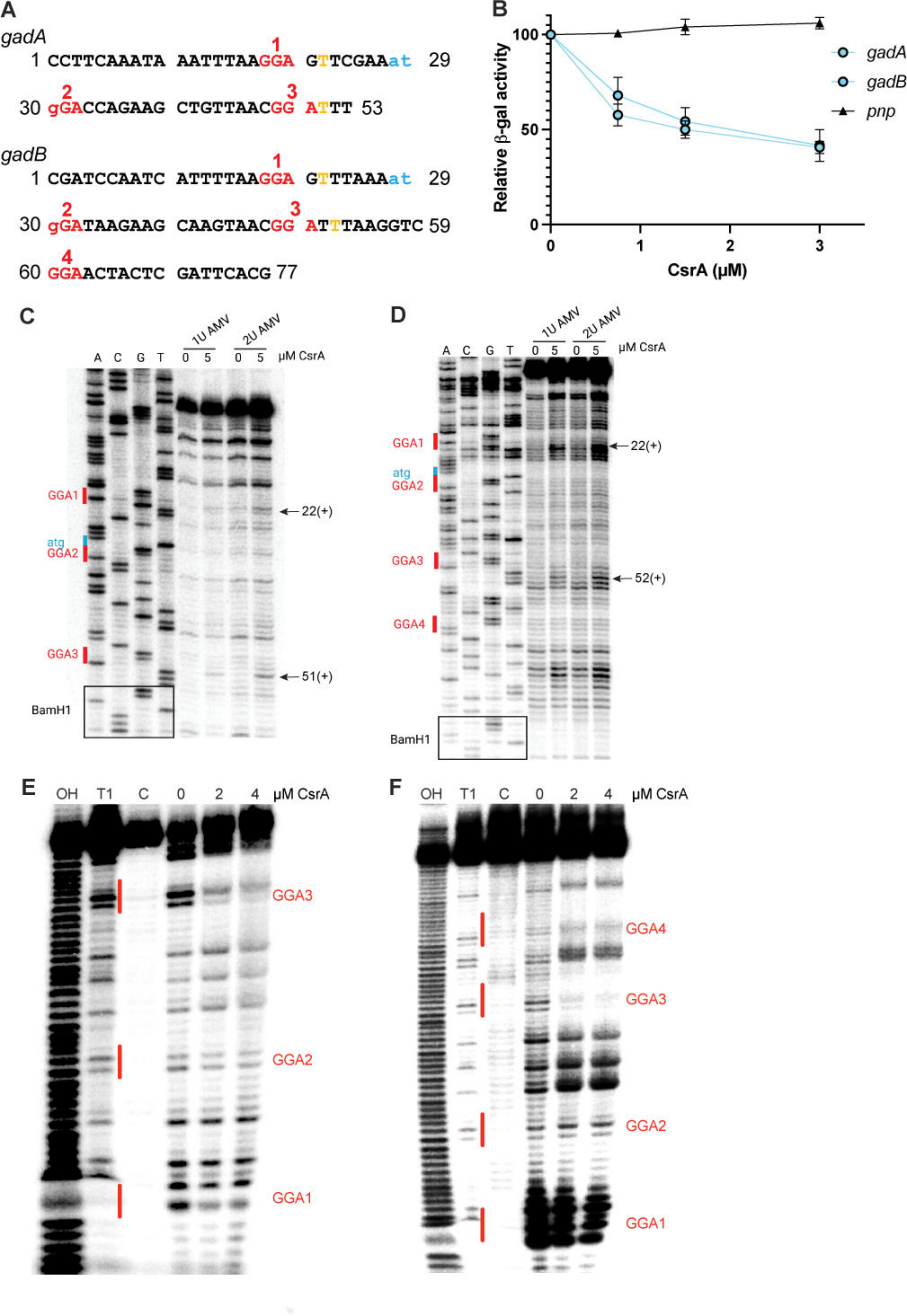
CsrA binding represses translation of *gadA* and *gadB in vitro*. (**A**) *gadA* and *gadB* leader sequences. The GGA motifs are shown in red and the start codons are in blue. Toeprint positions are indicated in yellow. (**B**) Effects of CsrA on *in vitro* translation of *gadA'-'lacZ*, *gadB'-'lacZ* and *pnp'-'lacZ* (control) translational fusions driven by a T7 RNAP promoter. Relative β-galactosidase activity depicts the mean and standard deviation relative to reaction mixtures lacking CsrA. (**C**) CsrA*-gadA* RNA toeprint using AMV reverse transcriptase. Toeprint positions are indicated on the right. (**D**) CsrA*-gadB* RNA toeprint using AMV reverse transcriptase. Toeprint positions are indicated on the right. (**E**) CsrA-*gadA* RNA footprint and (**F**) CsrA-*gadB* RNA footprint. 5′-end-labeled RNA was treated with RNase T1 ±CsrA as shown. Partial alkaline hydrolysis (OH) and RNase T1 digestion of denatured RNA (**T1**), and untreated control RNA are also shown.

We next examined the molecular mechanism of CsrA-dependent regulation of *gadA* and *gadB* expression. To determine whether CsrA repressed translation of *gadA* and *gadB*, we constructed plasmids with P_T7_-*gadA’-’lacZ* and P_T7_-*gadB’-’lacZ* translational fusions in which the 5′ leaders were fused to a T7 promoter and assessed their expression in the PURExpress system. In both cases, CsrA repressed *gadA* and *gadB*, indicative of direct translational repression (Fig. 9B).

We also performed RNA toeprinting and footprinting experiments to further investigate the regulatory mechanisms by which CsrA represses *gadA* and *gadB* translation. CsrA-dependent toeprints were observed just downstream of two GGA motifs in the *gadA* transcript. One GGA overlaps the *gadA* SD sequence (GGA1) and the second is in the early coding sequence (GGA3). A toeprint was not observed corresponding to GGA2 (Fig. 9A and C). In the case of *gadB*, CsrA-dependent toeprints were observed in regions that overlap the SD sequence (GGA1), and two in the early coding region (GGA3 and GGA4). A toeprint was not observed corresponding to GGA2 (Fig. 9A and D). To further examine the sites of CsrA binding, RNase T1 footprinting was performed. In both cases, this analysis suggested that the two most important CsrA interaction sites corresponded to GGA1, which overlaps the respective SD sequences, and GGA3, which is in the early coding sequences (Fig. 9E and F). These binding patterns are somewhat similar to CsrA-*sdiA* interaction, although CsrA binds exclusively within the coding region of *sdiA* (5).

### Acid shock does not trigger the expression of *csr* genes

Previous studies on the Csr system demonstrated that it is often subject to feedback loops in which CsrA represses the expression of genes involved in activating transcription of CsrB and CsrC sRNAs (2, 5, 7). Furthermore, while acid stress does not appear to affect CsrA transcription (50), the protonated forms of formate or acetate stimulate transcription of *csrB* and *csrC* by binding to the sensor-kinase BarA of the BarA-UvrY TCS which phosphorylates the transcription factor UvrY, which, in turn, activates *csrB/C* transcription (22). We therefore explored the effects of a pH shift from 7 to 5.5 on the expression of a *csrA’-‘lacZ* translational fusion, as well as on the expression of *csrB-lacZ* and *csrC-lacZ* transcriptional fusions in exponentially growing cells. A *ydeP’-‘lacZ* translational fusion served as a positive control, as *ydeP* expression increases in response to a decrease in pH (50, 51). The *ydeP’-‘lacZ* fusion showed the expected response (Fig. S6A). Expression of the *csrA’-‘lacZ* fusion was unaffected by the decrease in pH, consistent with previous observations on its transcription (Fig. S6B) (50). Expression of the *csrC-lacZ* fusion was not affected by the decrease in pH, whereas the *csrB-lacZ* showed a small increase in expression, starting after 25 min of exposure to acidic conditions (Fig. S6C and D). These results reveal that the key genes of the Csr system do not respond or respond weakly to acid stress.

### The *csrA* mutant alleviates GABA deficit in *Caenorhabditis elegans*

The *csrA* mutant overexpresses genes involved in the production of GABA, a neurotransmitter that regulates neuronal excitability in certain organisms including *C. elegans*. We investigated whether the *csrA* mutation would increase GABA production sufficiently to restore a GABA deficiency in *C. elegans* by assessing its effect on a GABA-dependent biological response. Nematodes harboring loss-of-function mutations in the *unc-25* gene are deficient in endogenous GABA synthesis and exhibit pronounced convulsions upon exposure to pentylenetetrazole (PTZ), a competitive GABA_A_ receptor antagonist that is used to chemically induce seizures to model epilepsy and other seizure disorders (68–71). Animals deficient in GABA lack the inhibitory signaling necessary to prevent the excitatory effects of PTZ on cholinergic signaling, thereby leading to PTZ-induced convulsions (71). Prior research demonstrated that exogenous GABA supplementation effectively restores normal phenotypes in *unc-25* mutants (72), and dietary utilization of a GABA-producing *E. coli* strain has neuroprotective effects in nematodes (73).

**Fig 10:**
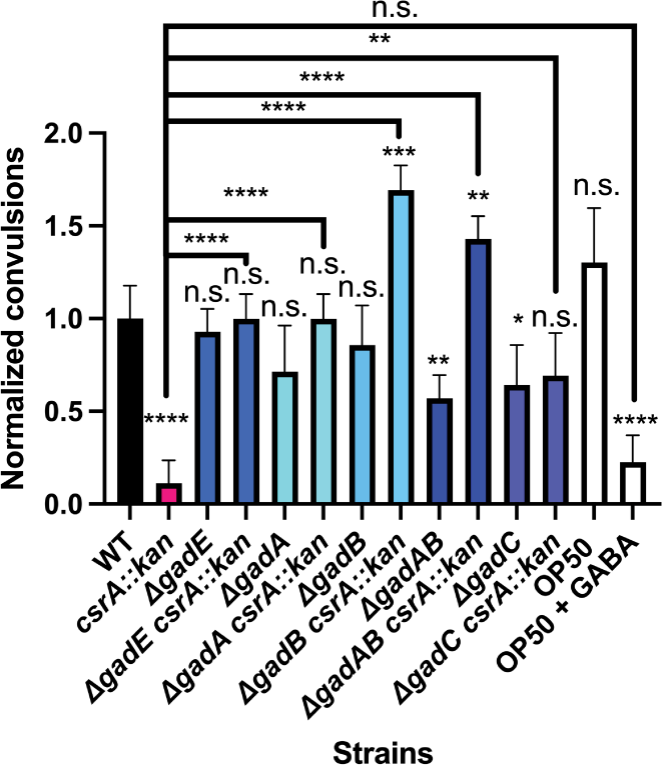
The effect of the *csrA* mutation on GABA-deficient *C. elegans*. Data are represented as the average percent of worms convulsing normalized to that in worms fed WT *E. coli* (MG1655) after 15 min of exposure to PTZ. Each data point is the average of a minimum of three independent experiments for a minimum of 60 worms. Error bars represent standard deviation (sd). Statistical significance was determined using unpaired *t*-tests and is denoted as follows: *P < 0.05; **P < 0.01; ***P < 0.001 ****P < 0.0001. The absence of a comparison bar indicates a comparison to WT.

After 15 min of PTZ exposure, the number of convulsing *unc-25* nematodes fed the WT *E. coli* strains OP50 or MG1655 was approximately fivefold higher than nematodes fed the *csrA* mutant derivative of MG1655 or those exogenously supplemented with GABA (Fig. 10). We used epistasis analysis to identify the genes responsible for the restorative effects of the *csrA* mutant on the GABA-deficient phenotype and discovered that deletion of either of the GABA producing glutamate decarboxylases, *gadA* or *gadB*, dampened the effects of the *csrA* mutant on convulsions (Fig. 10). Interestingly, the gene encoding the glutamate-GABA antiporter *gadC* appeared to have limited involvement in the restorative phenotype afforded by the *csrA* mutant, perhaps due to the activity of additional GABA transporters, such as the GABA permease YhiM (74). Together, these findings underscore the potential therapeutic relevance of *csrA* and bacterial GABA production in host disorders where GABA plays a critical biological role.

## DISCUSSION

Despite being a neutrophile, *E. coli* is capable of surviving extremely acidic conditions for extended periods of time through its expression of several acid resistance systems (31, 38). These systems are critical for survival in extremely acidic conditions but are detrimental to growth under mildly acidic conditions, presumably due to the metabolic drain that they exert (39, 48, 50, 75, 76). As a result, the acid stress systems are subject to complex and fine-tuned transcriptional regulatory circuits that are still not fully understood despite having been extensively studied (50, 55, 61, 77). In comparison, posttranscriptional regulation of the acid stress response has not been extensively studied, despite evidence suggesting that posttranscriptional regulation plays an important role in modulating the expression of many genes, which is the basis of CsrA-mediated regulation (3, 28, 36, 57, 78, 79).

We demonstrate that CsrA represses the EvgA-YdeO-GadE circuit of *E. coli* at multiple levels, from the top-tier regulator *evgA* to the structural genes *gadA* and *gadB*. Expression and activity of this circuitry are triggered by mildly acidic (pH 5–6) conditions, in anticipation of more extreme conditions (pH 1–3). CsrA is critical in preventing overexpression of the EvgA-YdeO-GadE circuit, and, in turn, preventing growth defects under mild acidity. CsrA deficiency results in severe growth defects under mildly acidic conditions and increased acid resistance in extremely acidic conditions, highlighting the critical role played by CsrA in balancing the trade-off between growth and survival.

Epistasis analysis showed that CsrA-dependent regulation of the EvgA-YdeO-GadE circuit was multitier in nature, and deleting *evgA* did not completely abolish the overexpression of *gadE’-‘lacZ* in a *csrA* mutant background (Fig. S5). Given that CsrA binds directly to *gadE* mRNA, the latter results were not surprising and demonstrate that CsrA represses *gadE* expression at more than one level, and not simply *via* effects on EvgA (Fig. 5B; Table S5).

High-affinity binding of CsrA to the *evgA*, *gadE*, *ydeO*, *gadB*, and *gadA* transcripts and *in vivo lacZ* reporter results also indicate that CsrA is involved in binding to and repressing multiple genes of the EvgA-YdeO-GadE circuit (Fig. 5, 7 and 9). The binding data are consistent with *in vitro* expression results, which demonstrated that CsrA directly repressed not only the top-tier regulator *evgA* but also the structural genes *gadA* and *gadB*. In the case of *gadA* and *gadB*, toeprinting showed that CsrA interacted with CsrA binding sites overlapping the SD sequence and the early coding sequences, which was supported by footprinting assays (Fig. 7 and 9). In the case of *evgA*, toeprinting and footprinting showed that CsrA interacted at multiple sites raising the possibility of a complex mechanism. This hypothesis is consistent with the results of *lacZ* reporter assays showing that translation of the leader peptide *evgL* is required for translation of *evgA*, implying that CsrA-dependent regulation of *evgA* involves translational coupling of the two genes, which is similar to CsrA-dependent regulation of *iraD* expression (64). However, unlike *iraD* where CsrA binds entirely upstream of the leader peptide coding sequence, CsrA binds both upstream and within the coding sequence of *evgL*. CsrA-dependent regulation of *evgLA* may require binding of a CsrA dimer to one site and then bridging to a second site in the segment preceding the *evgL* coding sequence as demonstrated previously (80). Doing so may enable another CsrA dimer to bind to a segment preceding *evgL* and to the site overlapping the *evgA* SD sequence*,* which is also within the coding region of *evgL*. This binding pattern would be consistent with gel shift results showing that CsrA binding to the 5′ leader of *evgA* results in at least two different shifts (Fig. 6). However, further analysis is required to fully elucidate the regulatory mechanism.

Interestingly, *gadA* but not *gadB* played an important role in the physiology of the *csrA* mutant under acidic conditions, affecting both growth and survival. These two isozymes were considered as interchangeable due to their high sequence similarity and similar phenotypes in the stationary phase (81). Complementing a *gadA* knockout with *gadB* restored the pH-dependent growth defect of the *csrA* mutant, suggesting that the combined level of GadA/B activity is more important for this growth phenotype than the particular isozyme (Fig. S4). This interpretation is consistent with the *lacZ* reporter results under acidic conditions in a *csrA* mutant background, where *gadA’-‘lacZ* was more highly expressed than *gadB’-‘lacZ* (Fig. 8). GadA also appears to play a role in survival under extremely acidic conditions without glutamate supplementation in exponentially growing cells. Previous work quantifying the amount of GadA and GadB produced in *E. coli* ATCC 11246, which is closely related to *E. coli* MG1655, showed that 80% of the isolated protein was GadA, explaining the disproportionate role of GadA in surviving extreme acidity (82, 83). While mutating *gadA* and *gadB* had differing effects on the physiology in a *csrA* mutant background, this pattern did not extend to the *C. elegans* studies. However, it is important to note that the latter studies were conducted on agar plates, where the cells were presumably in a stationary phase. Previous studies examining acid stress survival in the stationary phase noted no difference in the effects of *gadA* and *gadB*, suggesting that their distinct physiology may be limited to the exponential phase of growth (38, 84).

It is also interesting that reporter fusions for *csrA, csrB,* and *csrC* did not respond in a substantial way to acid stress induction, suggesting that expression of the Csr system itself is not strongly responsive to drops in pH. Despite this finding, we cannot definitively conclude that the Csr system is unresponsive to acid stress as the influence of CsrB/C RNA turnover was not examined in this study.

As CsrA is important for growth in *E. coli* and other species, it has become an appealing target for drug design (85–87). However, inhibiting CsrA causes increased biofilm formation and enhances the expression of virulence factors in some species, both of which are counterproductive for therapeutics (6, 88). The present study adds to the growing list of reasons why targeting CsrA for drug design may be counterproductive for antibiotic-based therapy of *E. coli* infections. Specifically, impairing CsrA-dependent regulation would greatly increase the expression of acid tolerance regulators of *E. coli*. This increase is relevant as acid tolerance has already been proposed to be involved in the low infectious dose of certain pathogenic *E. coli* (33). However, disrupting CsrA-dependent regulation of *gadA/B* may prove to be an appealing strategy for GABA production. GABA is a neurotransmitter that plays a key role in human mental health and microbiome research suggests that GABA-producing bacteria are inversely correlated with depression (44). Previous work explored engineering *E. coli* for batch production of GABA or even as a probiotic to deliver GABA directly; however, no previous studies have explored disrupting CsrA repression of *gadA/B* (89, 90). Our findings suggest that loss of repression of GDAR genes may be useful for increasing GABA levels in the host.

## MATERIALS AND METHODS

### Bacterial strains, plasmids, bacteriophage, culture conditions and oligonucleotides

All *E. coli* strains, plasmids, and bacteriophages used in this study are listed in Tables S2 to S4, respectively. Bacterial strains were grown and maintained in LB medium (0.5% yeast extract, 1% tryptone, and 1% NaCl, pH 7.4). Overnight cultures were inoculated in LB medium from frozen glycerol stocks of bacterial strains. These cultures were grown at 37°C or 30°C with shaking (250 rpm). Gene deletions were introduced by P1_vir_ transduction from *E. coli* donor strains from the Keio library (91). To remove antibiotic resistance cassettes, pCP20 encoding the Flp recombinase was used (92). Plasmids to complement knockouts were sourced from the ASKA collection (93).

M9 supplemented medium (1 × M9 salts supplemented with 2 mM MgSO_4_, 0.1 mM CaCl_2_, 0.2% casamino acids, and 0.4% glucose) was used for assessing growth and gene expression. For media used in survival assays, M9 was only supplemented with 0.4% glucose, and the pH was adjusted to pH 2.5 using HCl. When studying GDAR activity, growth media were supplemented with 1.5 mM glutamate. The pH of the medium was adjusted to 2.5 using HCl. For simulated stomach acid (pH 1.6), Fasted State Simulated Gastric Fluid (FaSSGF) Biorelevant medium was used.

Where appropriate, media contained the following antibiotics: ampicillin (100 µg/mL), tetracycline (15 µg/mL), kanamycin (100 µg/mL), and chloramphenicol (25 µg/mL). Oligonucleotide primers used in this study were synthesized by Integrated DNA Technologies.

Deletion of *csrA* results in severe growth defects and genetic instability (94). To avoid this problem, experiments were performed with strains carrying a *csrA* truncation (*csrA::kan*) that retains ~10% of its RNA binding activity (95). The *csrA::kan* allele was moved into various strains *via* P1 transduction (1).

### Construction of *lacZ* reporter fusions

Translational fusions to *‘lacZ* were constructed in plasmid vector pLFT (29). Posttranscriptional fusions to *‘lacZ* were constructed in plasmid vector placUV5 (29). All fusions were integrated into the chromosome using the CRIM system (96–98). Translational fusions were constructed as follows. About 500 nt of DNA upstream of the promoter region, the promoter region, and one or more codons downstream of the translation start site were amplified by PCR using the relevant primers (Table S3). Since *gadE* transcriptional regulation involves an extremely large upstream region, the translational fusion started ~750 nt upstream of the first promoter (67). The PCR products and pLFT were digested with PstI and BamHI and then ligated together. The products were transformed into DH5α λpir cells. The plasmids were isolated and fusion sequences were sequenced and verified before being integrated into the λ*att* site of strain MG1655 *ΔlacZ* using the helper plasmid pFINT as previously described (29). Single integrates were confirmed *via* PCR (96). Refer to Table S3 for the primer sequences.

The *evgA* leader fusion was constructed by PCR amplifying the 5′ leader of *evgA* (−114 to +7 relative to the first nucleotide of the start codon as +1) with primers that introduced restriction sites flanking the leader sequence. The resulting PCR product and pLacUV5 were digested with EcoRI and BamHI and then ligated together. To avoid the accumulation of spontaneous mutations that occurred when pLacUV5 plasmids were maintained in DH5α λpir cells, the ligated plasmids were integrated directly into the λatt site of MG1655 *ΔlacZ* using the Int_λ_ expressing helper plasmid pFINT (29). The resulting single integrates were confirmed by PCR and sequencing.

### β-galactosidase assay

Bacterial cultures containing *lacZ* fusions were grown in LB at 37°C to exponential phase (OD_600_ = 0.5), diluted to an optical density at 600 nm (OD_600_) of 0.02 in fresh M9 media supplemented with 0.4% glucose and 0.2% casamino acids. Cells were harvested at appropriate time points throughout growth during experiments. Acid induction experiments were based on a previously published protocol where a predetermined volume (1.6 mL) of 0.75 M HCl or water was added to actively growing cultures, HCl was added to decrease the pH from 7 to 5.5 and water was used as a negative control (50). β-Galactosidase activity was determined as described previously (29). Total cellular protein was measured following precipitation with 10% trichloroacetic acid, using the bicinchoninic acid (BCA) assay (Pierce Biotechnology) with bovine serum albumin as the protein standard.

### Growth curves and kinetics

Bacterial cultures were grown in LB at 37°C overnight and diluted to an OD_600_ of 0.01 in fresh M9 media. OD_600_ was measured every h for 8 h and at 24 h. The growth rate constant (μ) was calculated from the exponential phase of growth: μ = 2.303(logOD_2_ − logOD_1_)/(*t*_2_ – *t*_1_).

### Survival assays

Bacterial cultures were grown in LB at 37°C overnight and diluted to OD_600_ of 0.01 in fresh M9 media. Cells were grown to an OD_600_ of 0.5, pelleted by centrifugation, and then washed with a M9 medium containing 0.4% glucose. The wash medium was the same pH as the growth media and contained no added amino acids. Cells were then diluted 1:50 into pH 2.5 M9 medium containing 0.4% glucose with or without 1.5 mM glutamate and incubated for 2 h without shaking at 37°C. Aliquots were serially diluted in triplicate and were plated onto LB agar plates. Colonies were counted after being grown overnight at 37°C. Percent survival was calculated as follows: ((CFU per mL at 2 h) / (CFU per mL at 0 h))∗100.

### Gel shift assays

Binding of CsrA to the 5′ leader of *gadA* (60 nt; −27 to +33 relative to the first nucleotide of the start codon as +1), *gadB* (73 nt; −27 to +43 relative to the start codon), *gadE* (28 nt; −21 to +7 relative to the start codon), *evgA* (132 nt; −124 to +8 relative to the start codon), *ydeO* (84 nt; −74 to +10 relative to the start codon), and *ydeP* (188 nt; −178 to +10 relative to the start codon) was monitored using a gel shift assay. The transcripts were synthesized *in vitro* using the MEGAshortscript Kit (Invitrogen). Templates for *in vitro* transcription reactions were generated by PCR and subjected to PCR cleanup using Monarch PCR & DNA Clean up Kit (New England BioLabs, NEB) before using for *in vitro* transcription reactions. *In vitro* synthesized transcripts were gel purified on denaturing urea polyacrylamide gels, eluted overnight, extracted with phenol-chloroform, ethanol precipitated, resuspended in TE buffer, and quantified with a spectrophotometer. Twenty pmol of the RNA was dephosphorylated with Antarctic phosphatase (NEB) and 5′-end-labeled with γ-^32^P ATP and T4 polynucleotide kinase (NEB), gel purified, eluted overnight, phenol-chloroform extracted, ethanol precipitated, and resuspended in TE. The concentration of labeled RNA was determined using a standard curve constructed with γ-^32^P ATP. Binding reactions contained 0.08–0.2 nM end-labeled RNA, 10 mM MgCl_2_, 100 mM KCl, 32.5 ng SUPER-ase In (Ambion) with various concentrations of CsrA-H6 as indicated in the figures and incubated at 37°C for 30 min (99). Reaction mixtures were separated on a native polyacrylamide gel and imaged using a phosphorimager. The radioactive signals of free and shifted/bound RNA-protein complexes were quantified with Quantity One software and used for determining the apparent equilibrium dissociation constants (K_D_).

### Coupled transcription-translation assays

Coupled transcription-translation assays were conducted *in vitro* using the PURExpress kit (NEB) following a published protocol (12). Plasmid pGadA-T7 contains a T7 promoter to drive transcription of the *gadA* translational fusion (nt +1 to +54 relative to the transcriptional start site). Plasmid pGadB-T7 contains a T7 promoter to drive transcription of the *gadB* translational fusion (nt +1 to +78 relative to the transcriptional start site). Plasmid pEvgA-T7 contains a T7 promoter to drive transcription of the *evgA* translational fusion (nt +1 to +122 relative to the transcriptional start site). The transcription start site of the σ^S^ promoter was chosen over the sigma σ^70^ promoter due to previous results suggesting it may be more active (66). A similar P_T7_-*pnp'-'lacZ* translational fusion plasmid was used as a negative control (65). These plasmids were used as templates for coupled transcription-translation reactions according to the manufacturer’s instructions. Each 6.7 µL reaction contained 250 ng of plasmid DNA and various concentrations of purified CsrA-His6 with 1 U of RNase inhibitor (Promega) and 2.5 mM dithiothreitol (DTT), 2.7 µL of solution A and 2 µL of solution B. The mixtures were incubated for 2 h at 37°C and β-galactosidase activity was determined. OD_420_ data without CsrA were normalized to 100%.

### Toeprint assays

CsrA-RNA toeprint assays followed a previously published procedure (19). *gadA* RNA (from nt −27 to +26 relative to the *gadA* translational start), *gadB* RNA (nt −27 to +50 relative to the *gadB* translational start), and *evgA* RNA (nt −114 to +8 relative to the *evgA* translational start) were synthesized with the RNAMaxx kit (Agilent technologies) using PCR-generated DNA templates. Each gel-purified RNA (150 nM) in TE buffer was hybridized to a 5′ end-labeled DNA oligonucleotide complementary to the 3′ end of the vector-derived 3′ extension by heating for 3 min at 80°C followed by slow cooling for 10 min at room temperature. Toeprint reaction mixtures (10  µL) contained 2  µL of the hybridization mixture (30  nM final concentration), 1 µM CsrA-H6, 375 µM each dNTP, 10  mM DTT and Superscript III (SSIII), or AMV reverse transcriptase buffer. Mixtures were incubated for 30 min at 37°C to allow CsrA-RNA complex formation. After the addition of 0.125–0.25 U SSIII (Invitrogen) or 0.5–2 U AMV reverse transcriptase (Sigma Aldrich) incubation was continued for 15 min at 37°C. Reactions were terminated by the addition of 10 µL of gel loading buffer (95% formamide, 0.025% SDS, 20  mM EDTA, 0.025% bromophenol blue, and 0.025% xylene cyanol). Samples were heated to 90°C for 5 min and fractionated through standard 6% polyacrylamide-8 M urea sequencing gels. Toeprint patterns were visualized with a phosphorimager.

### Footprint assays

CsrA footprints were performed according to the published procedure (95). The *gadA, gadB,* and *evgA* RNAs described for the toeprint assay were used for footprinting. Gel-purified RNA was dephosphorylated and then 5′ end-labeled using T4 polynucleotide kinase (NEB) and [γ−32P]ATP (7,000  Ci/mmol). Labeled RNAs were renatured by heating for 1  min at 90°C followed by slow cooling for 10 min at room temperature. Binding reaction mixtures (10  µL) contained 2  nM labeled RNA, 10  mM Tris-HCl (pH 7.5), 10  mM MgCl_2_, 100  mM KCl, 40  ng of yeast RNA, 7.5% glycerol, 0.1  mg/mL xylene cyanol, and various concentrations of purified CsrA-H6. After a 30-min incubation at 37°C to allow for CsrA-RNA complex formation, RNase T1 (0.016 U) was added, and the incubation was continued for 15  min at 37°C. The reactions were stopped by adding 10  µL of gel loading buffer. Samples were heated for 5  min at 90°C and fractionated through standard 6% polyacrylamide-8 M urea sequencing gels. Cleaved patterns were examined using a phosphorimager.

### *C. elegans* studies

Nematodes were maintained as previously described (100, 101). The *C. elegans* strains used in this study, CB156 (*unc-25*) and N2 (Bristol), were obtained from the Caenorhabditis Genetics Center. Bacterial strains used in all *C. elegans* experiments were grown overnight in Lennox Broth (LB) at 37°C with shaking, seeded onto nematode growth media (NGM), and dried in a biosafety cabinet for 2–4 h. Worms were age-synchronized using the standard sodium hypochlorite method. Age-synchronized worms were plated onto NGM seeded with appropriate bacteria and were kept at 23°C for 2 days. Positive control plates were bathed in 30 mM GABA solution (102) for 3 h prior to starting the experiments. Worms were picked and placed on NGM plates supplemented with PTZ (7 mg/mL) for 15 min (68). Following the incubation, the number of worms displaying a convulsing (head bobbing) phenotype was counted. As a control, PTZ-treated N2 worms did not display any convulsions, consistent with previous reports (68).

### Datamining methods

Hypothetical targets of CsrA-dependent regulation were selected from previously published studies. Evidence for CsrA affecting transcript levels was determined by examining RNA-seq data (3, 24, 28). Genes were considered hypothetical targets if the log_2_ fold change was >1 or < −1 and was statistically significant. Potential RNA targets derived from pulldown results (29), *in silico* prediction (30), CLIP-seq (3), and HITS-CLIP-seq (28) were obtained from previously published studies. The data were compiled and are represented in Table S1; Fig. 1.

## Data Availability

The authors confirm all supporting data, code, and protocols have been provided within the article or through supplementary data files.

## SUPPLEMENTAL MATERIAL

Supplementary Information jb.00354-23-s0001.docx Tables S1 to S4 and Figures S1 to S6.

ASM does not own the copyrights to Supplemental Material that may be linked to, or accessed through, an article. The authors have granted ASM a non-exclusive, world-wide license to publish the Supplemental Material files. Please contact the corresponding author directly for reuse.
