# Supplementary material for: Multitier regulation of the E. coli extreme acid stress response by CsrA
Source: J Bacteriol. 2024 Feb 6;206(4):e00354-23. doi: 10.1128/jb.00354-23 (PMC11210196; doi:10.1128/jb.00354-23)
Supplement: Supplementary Information — Tables S1 to S4 and Figures S1 to S6. [file jb.00354-23-s0001.docx]

**Table S1. Effects of CsrA on the expression of acid stress genes found in previous studies**

**Fold change^D^**

**Gene Pulldown^A^ *In silico*^B^ CLIP-seq^C^ HITS-seq^C^ (24) (3) (28)**

*evgA* **–**^E^ **+** **–** **+** 0.66 0.55 0.97

*evgS* **–** **–** **–** **–** 0.43 0.17 1.04

*ydeP* **–** **–** **–** **–** 1.64 0.42 2.99

*ydeO* **–** **–** NA^F^ **–** 1.10 NA 1.29

*gadE* **–** **+** NA **+** 1.20 NA 1.29

*gadA* **+**^E^ **+** **–** **+** 3.16 1.24 0.54

*gadB* **–** **+** **–** **+** 3.50 1.73 0.37

*gadC* **–** **–** **–** **+** 3.40 0.99 0.31

^A^ Pulldown refers to CsrA bound RNAs (29).

^B^*In silico* predictions of CsrA binding sites (30).

^C^CLIP-seq (3) and HITS-CLIP-seq (28) refer to CsrA binding targets identified by these methods.

^D^RNA-seq studies include Log_2_ fold changes >1 (3, 24, 28).

^E^ –, indicates no measured/predicted interaction; +, indicates a measured/predicted interaction.

^F^NA, transcripts for the gene were not measured.

**Table S2. *E. coli* and nematode strains**

**Strain Genotype Source**

MG1655 F^−^ λ^−^ *rph*-*1*

OP50 *ura-*  (103)

AP437 MG1655 / Δ*lacZ* (98)

TRMG1655 MG1655 / *csrA*::*kan* (1)

BW25113 *Δ(araD-araB)567 Δ(rhaD-rhaB)568*  (92)

*ΔlacZ4787*(::rrnB-3) *hsdR514 λ^-^ rph-1*

BW25113 *ΔevgA::kan* BW25113 / marked *evgA* deletion Km^r^  (92)

BW25113 Δ*gadE::kan* BW25113 / marked *gadE* deletion Km^r^ (92)

BW25113 *ΔgadA::kan* BW25113 / marked *gadA* deletion Km^r^ (92)

BW25113 *ΔgadB::kan* BW25113 / marked *gadB* deletion Km^r^  (92)

BW25113 *ΔgadC::kan* BW25113 / marked *gadC* deletion Km^r^ (92)

BW25113 *ΔevgS::kan* BW25113 / marked *evgS* deletion Km^r^ (92)

BW25113 *ΔydeO::kan* BW25113 / marked *ydeO* deletion Km^r^ (92)

BW25113 *ΔydeP::kan* BW25113 / marked *ydeP* deletion Km^r^ (92)

AP461 MG1655 Δ*lacZ*/pLFXcsrB-*lacZ* (98)

AP858 MG1655 Δ*lacZ*/pLFXcsrC-*lacZ* (98)

PLB1424 AP437 / P1–P5*–csrA′–′lacZ* Ap^r^ (19)

MGG01 MG1655 / *ΔgadE::kan* Km^r^ This study

MGG02 MG1655 / *ΔgadE csrA::kan* Km^r^  This study

MGG03 MG1655 / *ΔgadA::kan* Km^r^ This study

MGG04 MG1655 / *ΔgadA csrA::kan* Km^r^ This study

MGG05 MG1655 / *ΔgadB::kan* Km^r^ This study

MGG06 MG1655 / *ΔgadB csrA::kan* Km^r^ This study

MGG07 MG1655 / *ΔgadC::kan* Km^r^ This study

MGG08 MG1655 / *ΔgadC csrA::kan* Km^r^  This study

MGG09 MG1655 / *ΔgadA::kan ΔgadB* Km^r^ This study

MGG10 MG1655 / *ΔgadA ΔgadB csrA::kan* Km^r^ This study

MGG11 MG1655 / *ΔevgA::kan* Km^r^ This study

MGG12 MG1655 / *ΔevgA csrA::kan* Km^r^  This study

MGG13 MG1655 / *ΔevgS::kan* Km^r^ This study

MGG14 MG1655 / *ΔevgS csrA::kan* Km^r^ This study

MGG15 MG1655 / *ΔydeO::kan* Km^r^  This study

MGG16 MG1655 / *ΔydeO csrA::kan* Km^r^  This study

MGG17 MG1655 / *ΔydeP::kan* Km^r^ This study

MGG18 MG1655 / *ΔydeP csrA::kan* Km^r^  This study

MGG19 MG1655 / pBR322 Tc^r^ Ap^r^ This study

MGG20 MG1655 / p2VR112 Ap^r^ This study

MGG21 MG1655 / *csrA::kan* pBR322 Tc^r^ Ap^r^ Km^r^  This study

MGG22 MG1655 / *csrA::kan* p2VR112 Ap^r^ Km^r^ This study

MGG23 MG1655 / *ΔgadE::kan* pCA24N Km^r^ Cm^r^ This study

MGG24 MG1655 / *ΔgadE csrA::kan* pCA24N Km^r^ Cm^r^ This study

MGG25 MG1655 / *ΔgadE::kan* pCA-*gadE* Km^r^ Cm^r^ This study

MGG26 MG1655 / *ΔgadE csrA::kan* pCA-*gadE* Km^r^ Cm^r^ This study

MGG27 MG1655 / *ΔgadA::kan* pCA24N Km^r^ Cm^r^ This study

MGG28 MG1655 / *ΔgadA csrA::kan* pCA24N Km^r^ Cm^r^ This study

MGG29 MG1655 / *ΔgadA::kan* pCA-*gadA* Km^r^ Cm^r^ This study

MGG30 MG1655 / *ΔgadA csrA::kan* pCA-*gadA* Km^r^ Cm^r^ This study

MGG31 MG1655 / *ΔgadB::kan* pCA24N Km^r^ Cm^r^ This study

MGG32 MG1655 / *ΔgadB csrA::kan* pCA24N Km^r^ Cm^r^ This study

MGG33 MG1655 / *ΔgadB::kan* pCA-*gadB* Km^r^ Cm^r^ This study

MGG34 MG1655 / *ΔgadB csrA::kan* pCA-*gadB* Km^r^ Cm^r^ This study

MGG35 MG1655 / *ΔgadC::kan* pCA24N Km^r^ Cm^r^ This study

MGG36 MG1655 / *ΔgadC csrA::kan* pCA24N Km^r^ Cm^r^ This study

MGG37 MG1655 / *ΔgadC::kan* pCA-*gadC* Km^r^ Cm^r^ This study

MGG38 MG1655 / *ΔgadC csrA::kan* pCA-*gadC* Km^r^ Cm^r^ This study

MGG39 MG1655 / *ΔgadA::kan ΔgadB* pCA24N Km^r^ Cm^r^ This study

MGG40 MG1655 / *ΔgadA ΔgadB csrA::kan* pCA24N Km^r^ Cm^r^ This study

MGG41 MG1655 / *ΔgadA::kan ΔgadB* pCA-*gadA* Km^r^ Cm^r^ This study

MGG42 MG1655 / *ΔgadA ΔgadB csrA::kan* pCA-*gadA* Km^r^ Cm^r^ This study

MGG43 MG1655 / *ΔgadA::kan ΔgadB* pCA-*gadB* Km^r^ Cm^r^ This study

MGG44 MG1655 / *ΔgadA ΔgadB csrA::kan* pCA-*gadB* Km^r^ Cm^r^ This study

MGG45 MG1655 / *ΔevgA::kan* pCA24N Km^r^ Cm^r^ This study

MGG46 MG1655 / *ΔevgA csrA::kan* pCA24N Km^r^ Cm^r^ This study

MGG47 MG1655 / *ΔevgA::kan* pCA-*evgA* Km^r^ Cm^r^ This study

MGG48 MG1655 / *ΔevgA csrA::kan* pCA-*evgA* Km^r^ Cm^r^ This study

MGG49 MG1655 / *ΔevgS::kan* pCA24N Km^r^ Cm^r^ This study

MGG50 MG1655 / *ΔevgS csrA::kan* pCA24N Km^r^ Cm^r^ This study

MGG51 MG1655 / *ΔevgS::kan* pCA-*evgS* Km^r^ Cm^r^ This study

MGG52 MG1655 / *ΔevgS csrA::kan* pCA-*evgS* Km^r^ Cm^r^ This study

MGG53 MG1655 / *ΔydeO::kan* pCA24N Km^r^ Cm^r^ This study

MGG54 MG1655 / *ΔydeO csrA::kan* pCA24N Km^r^ Cm^r^ This study

MGG55 MG1655 / *ΔydeO::kan* pCA-*ydeO* Km^r^ Cm^r^ This study

MGG56 MG1655 / *ΔydeO csrA::kan* pCA-*ydeO* Km^r^ Cm^r^ This study

MGG57 MG1655 / *ΔydeP::kan* pCA24N Km^r^ Cm^r^ This study

MGG58 MG1655 / *ΔydeP csrA::kan* pCA24N Km^r^ Cm^r^ This study

MGG59 MG1655 / *ΔydeP::kan* pCA-*ydeP* Km^r^ Cm^r^ This study

MGG60 MG1655 / *ΔydeP csrA::kan* pCA-*ydeP* Km^r^ Cm^r^ This study

MGG61 AP437 / pLFT*evgA'-'lacZ pgaC::cam* Ap^r^ Cm^r^ This study

MGG62 AP437 / pLacUV5*evgA'-'lacZ pgaC::cam* Ap^r^ Cm^r^ This study

MGG63 AP437 / pLFT*evgA'-'lacZ pgaC::cam csrA::kan* Ap^r^ Cm^r^ Km^r^  This study

MGG64 AP437 / pLacUV5*evgA'-'lacZ pgaC::cam csrA::kan*  This study

Ap^r^ Cm^r^ Km^r^

MGG65 AP437 / pLFT*gadA'-'lacZ pgaC::cam* Ap^r^ Cm^r^ This study

MGG66 AP437 / pLFT*gadA'-'lacZ pgaC::cam csrA::kan* Ap^r^ Cm^r^ Km^r^  This study

MGG67 AP437 / pLFT*gadB'-'lacZ pgaC::cam* Ap^r^ Cm^r^ This study

MGG68 AP437 / pLFT*gadB'-'lacZ pgaC::cam csrA::kan* Ap^r^ Cm^r^ Km^r^  This study

MGG69 AP437 / pLacUV5*gadB'-'lacZ pgaC::cam csrA::kan* This study

Ap^r^ Cm^r^ Km^r^

MGG70 AP437 / pLFT*ydeO'-'lacZ pgaC::cam* Ap^r^ Cm^r^ This study

MGG71 AP437 / pLFT*ydeO'-'lacZ pgaC::cam csrA::kan* Ap^r^ Cm^r^ Km^r^  This study

MGG72 AP437 / pLFT*ydeP'-'lacZ pgaC::cam* Ap^r^ Cm^r^ This study

MGG73 AP437 / pLFT*ydeP'-'lacZ pgaC::cam csrA::kan* Ap^r^ Cm^r^ Km^r^  This study

MGG74 AP437 / pLFT*gadE'-'lacZ pgaC::cam* Ap^r^ Cm^r^ This study

MGG75 AP437 / pLFT*gadE'-'lacZ pgaC::cam ΔevgA::kan* Ap^r^ Cm^r^ Km^r^  This study

MGG76 AP437 / pLFT*evgA*(A89T T90A)*'-'lacZ pgaC::cam* Ap^r^ Cm^r^ This study

MGG77 AP437 / pLFT*evgA*(A89T T90A)*'-'lacZ pgaC::cam csrA::kan*  This study

Ap^r^ Cm^r^ Km^r^

MGG78 AP437 / pLFT*evgL'-'lacZ* Ap^r^ This study

MGG79 AP437 / pLFT*evgL'-'lacZ csrA::kan* Ap^r^ Km^r^ This study

*C. elegans* N2 Bristol Caenorhabditis

genetics center

*C. elegans* CB156 *unc-25* Caenorhabditis genetics center

**Table S3. Primer sequences**

**Name Sequence (5'-3') Purpose**

*evgA* conf fwd ATGAGATGACGCCTTATGTC Primer to confirm

*evgA* deletion

*evgA* conf rev CGAAACTTATGGTCGACCAA Primer to confirm

*evgA* deletion

*gadE* conf fwd CGATTCGGACAAGGATGTAA Primer to confirm

*gadE* deletion

*gadE* conf rev GCCTCTCCTTTAGTAATCACC Primer to confirm

*gadE* deletion

*evgS* conf fwd GAATGTAAATCACTGATGGATCT Primer to confirm

*evgS* deletion

*evgS* conf rev GCCAGGAGTTCTCATCATAAAT Primer to confirm

*evgS* deletion

*ydeO* conf fwd TGCAAGAGATCTTACACATTTC Primer to confirm

*ydeO* deletion

*ydeO* conf rev TTTGCTAACGAGTAGTCAAC Primer to confirm

*ydeO* deletion

*gadA* conf fwd GCCTTGCTTCCATTGCGG Primer to confirm

*gadA* deletion

*gadA* conf rev TAAAGGCTGGGCATTCGG Primer to confirm

*gadA* deletion

*gadB* conf fwd TAAACACGAGTCCTTTGCAC Primer to confirm

*gadB* deletion

*gadB* conf rev CCAGTGAACAGACTTTGGAA Primer to confirm

*gadB* deletion

*gadC* conf fwd GTCTGTTCACTGGCATTAG Primer to confirm

*gadC* deletion

*gadC* conf rev CAATACGACAGCAAGCAC Primer to confirm

*gadC* deletion

*evgA-'lacZ* fwd GAGGAGCTGCAGCGACAGTGACACTACCTG Primer to construct

translational fusion

*evgA-'lacZ* rev CTCCTCGGATCCGCGTTCATAGATTATTCCCT Primer to construct

translational fusion

*ydeO-'lacZ* fwd GAGGAGCTGCAGCAACGAGATGTGGTCTTTA Primer to construct

translational fusion

*ydeO-'lacZ* rev CTCCTC GGATCCCTGTGGTCGCATGCATTTC Primer to construct

translational fusion

*ydeP-'lacZ* fwd GAGGAGCTGCAGTGACAGTAGCCGACAAGTG Primer to construct

translational fusion

*ydeP-'lacZ* rev CTCCTCGGATCCGCAGCACCCTGGTAGGATTC Primer to construct

translational fusion

*gadA-'lacZ* fwd GAGGAGCTGCAGGACGTTCCTTTGAACCGTTG Primer to construct

translational fusion

*gadA-'lacZ* rev CTCCTCGGATCCAAATCCGTTAACAGCTTCTG Primer to construct

GTC translational fusion

*gadE-'lacZ* fwd GAGGAGCTGCAGCGGATCATAGTCACTTGATG Primer to construct

translational fusion

*gadE-'lacZ* rev CTCCTCGGATCCATCATAACTTGCTCCTTAGCC Primer to construct

translational fusion

*gadB-'lacZ* fwd GAGGAGGAATTCCGATAAATCCTACTTTTTTA Primer to construct

ATGCGATCC translational fusion

*gadB-'lacZ* rev CTCCTCGGATCCCGTGAATCGAGTAGTTCCG Primer to construct

ACC translational fusion

*evgA-'lacZ* GAGGAGGAATTCATTATCTTAAAGGAAGCTC Primer to construct

pLacUV5 fwd AGATTTTC leader fusion

*evgL* 89 90 codon fwd TACTACAGGGAGAAGGGAGTAGCTTCATTGC Primer for point mutations

AAAGGGAAT

*evgL* 89 90 codon rev ATTCCCTTTGCAATGAAGCTACTCCCTTCTCCC Primer for point mutations

TGTAGTA

*evgA* EMSA fwd TAATACGACTCACTATAGGGCTTGTCGAATTA Primer to generate *in vitro* CTTAAAGG transcription template

*evgA* EMSA rev GCGTTCATAGATTATTCCC Primer to generate *in vitro*

transcription template

*gadA* EMSA fwd TAATACGACTCACTATAGGGCCTTCAAATAAA Primer to generate *in vitro*

TTTAAGG transcription template

*gadA* EMSA rev TGAGCGGAAATCCGTTAAC Primer to generate *in vitro*

transcription template

*gadB* EMSA fwd TAATACGACTCACTATAGGGCGATCCAATCAT Primer to generate *in vitro*

TTTAAGG transcription template

*gadB* EMSA rev CGAGTAGTTCCGACCTTA Primer to generate *in vitro*

transcription template

*gadE* EMSA fwd TAATACGACTCACTATAGGGACGTATTAGTTC Primer to generate *in vitro*

ACGAAG transcription template

*gadE* EMSA rev AAATCATAACTTGCTCCTTAG Primer to generate *in vitro*

transcription template

*ydeO* EMSA fwd TAATACGACTCACTATAGGGAAACAAACAGC Primer to generate *in vitro*

AAATTATAAATATG transcription template

*ydeO* EMSA rev CGAGCGACATTTTATCTCC Primer to generate *in vitro*

transcription template

*ydeP* EMSA fwd TAATACGACTCACTATAGGGATGAGAGATGA Primer to generate *in vitro*

TGCTTAACTTATCG transcription template

*ydeP* EMSA rev TTTTCTTCATCTACTTATCCTGTGTG Primer to generate *in vitro*

transcription template

T7 rev CCCTATAGTGAGTCGTATTAGCTGCAGGCAT Primer to replace native GCAAGCTTG promoter with T7 promoter

*evgA* T7 fwd CTAATACGACTCACTATAGGGATTATCTTAAA Primer to replace native

GGAAGCTC promoter with T7 promoter

*gadA* T7 fwd CTAATACGACTCACTATAGGGCCTTCAAATAA Primer to replace native ATTTAAGG promoter with T7 promoter

*gadB* T7 fwd CTAATACGACTCACTATAGGGCGATCCAATCA Primer to replace native

promoter with T7 promoter

**Table S4. Plasmids**

**Plasmid Description Source**

pLacUV5 Used for constructing leader fusions Ap^r^ (29)

pLFT Used for constructing translational fusions Ap^r^ (29)

pPFINT Helper plasmid used for integrating *lacZ* fusions into the chromosome Tc^r^ (29)

pCP20 Used for eliminating antibiotic resistance genes Cm^r^ (93)

pBR322 Cloning vector Ap^r^ Tc^r^ (99)

p2VR112 *csrA* cloned into pBR322 Ap^r^ (26)

pCA24N Cloning vector Cm^r^ (94)

pCA-*gadE* IPTG-inducible *gadE* overexpression vector from the ASKA library Cm^r^ (94)

pCA-*gadA* IPTG-inducible *gadA* overexpression vector from the ASKA library Cm^r^ (94)

pCA-*gadB* IPTG-inducible *gadB* overexpression vector from the ASKA library Cm^r^ (94)

pCA-*gadC* IPTG-inducible *gadC* overexpression vector from the ASKA library Cm^r^ (94)

pCA-*evgA* IPTG-inducible *evgA* overexpression vector from the ASKA library Cm^r^ (94)

pCA-*evgS* IPTG-inducible *evgS* overexpression vector from the ASKA library Cm^r^ (94)

pCA-*ydeO* IPTG-inducible *ydeO* overexpression vector from the ASKA library Cm^r^ (94)

pCA-*ydeP* IPTG-inducible *ydeP* overexpression vector from the ASKA library Cm^r^ (94)

pLFT-T7*evgA'-'lacZ* *evgA'-'lacZ* translational fusion in pLFT for PURExpress Ap^r^ This study

pLFT-T7*gadA'-'lacZ* *gadA'-'lacZ* translational fusion in pLFT for PURExpress Ap^r^ This study

pLFT-T7*gadB'-'lacZ* *gadB'-'lacZ* translational fusion in pLFT for PURExpress Ap^r^ This study

pT7-*pnpFL'-'lacZ* *pnpFL'-'lacZ* translational fusion for PURExpress (negative control) Ap^r^  (12)

**
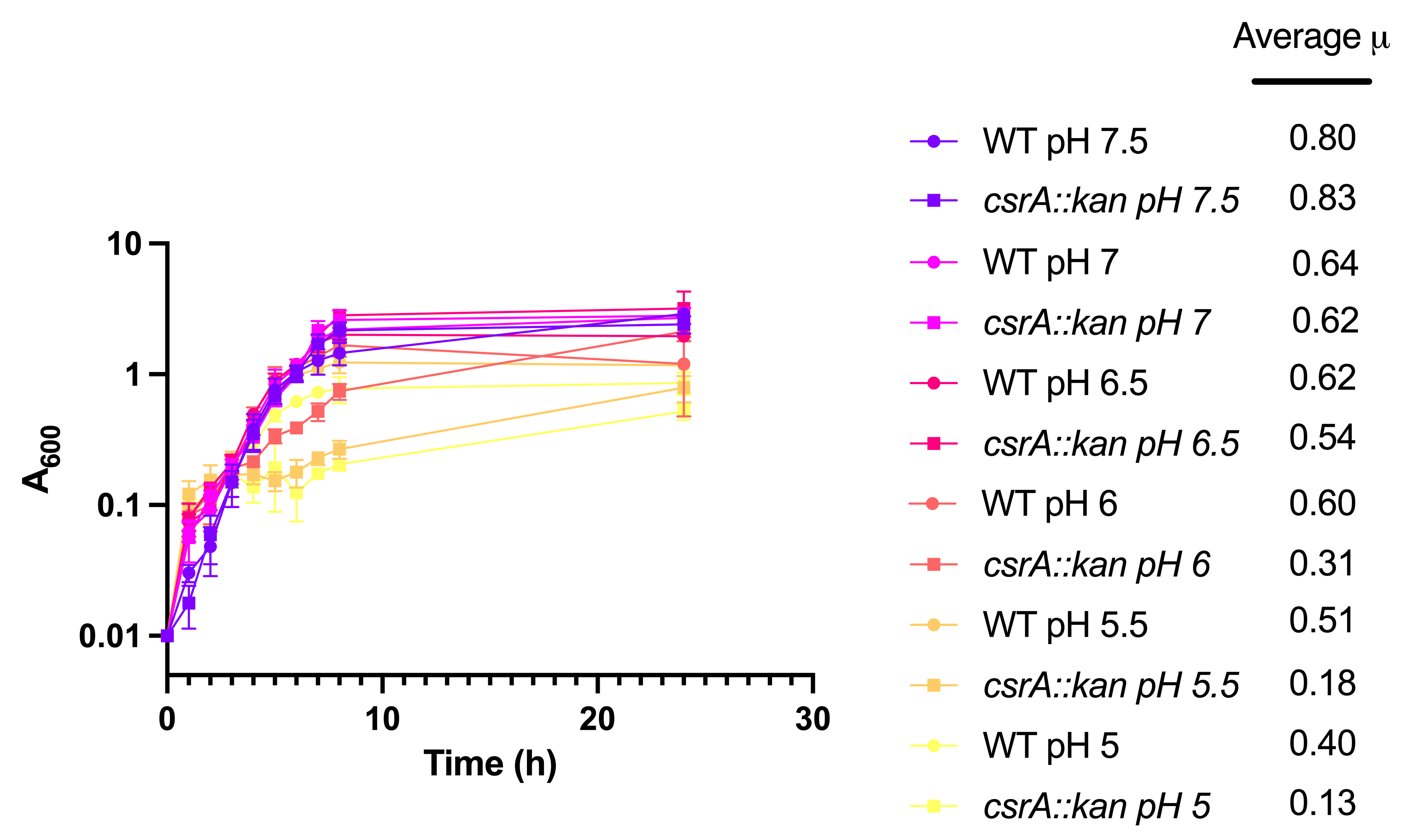
**

**FIG S1** Growth across pH conditions. Cells were grown in M9 minimal media adjusted to different pH values. The pH is color coded. Circles, wild type (WT); squares; *csrA::kan* mutant. μ, growth rate. Error bars represent standard deviation (sd) from four independent experiments.

**
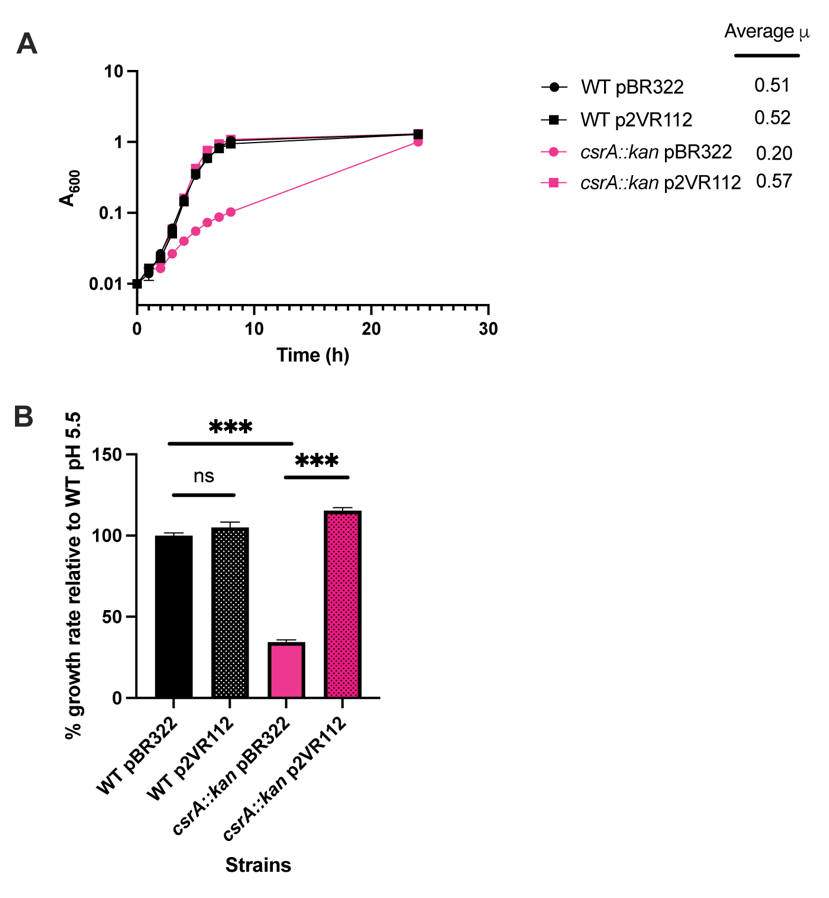
**

**FIG S2** Complementing *csrA::kan* abolishes the pH-dependent growth defect. (A) Growth curves and growth rates (μ) of exponential phase *E. coli* strains in mildly acidic (pH 5.5) M9 media. (B) Growth rates of exponential phase *E. coli* strains in mildly acidic (pH 5.5) M9 media normalized to WT. Error bars represent standard deviation (sd) from three independent experiments. Statistical significance was determined using unpaired *t* tests and is denoted as follows: ***, P<.001. ns, not significant.

**FIG S3** Suppressors of the pH-dependent growth defect in the *csrA* mutant. (A-H) Growth curves of exponential phase *E. coli* strains in mildly acidic (pH 5.5) M9 media. Gene products of possible suppressors are indicated in the heading of each panel. Average growth rate (μ) of each strain is shown on the right. Error bars represent standard deviation (sd) from six independent experiments.


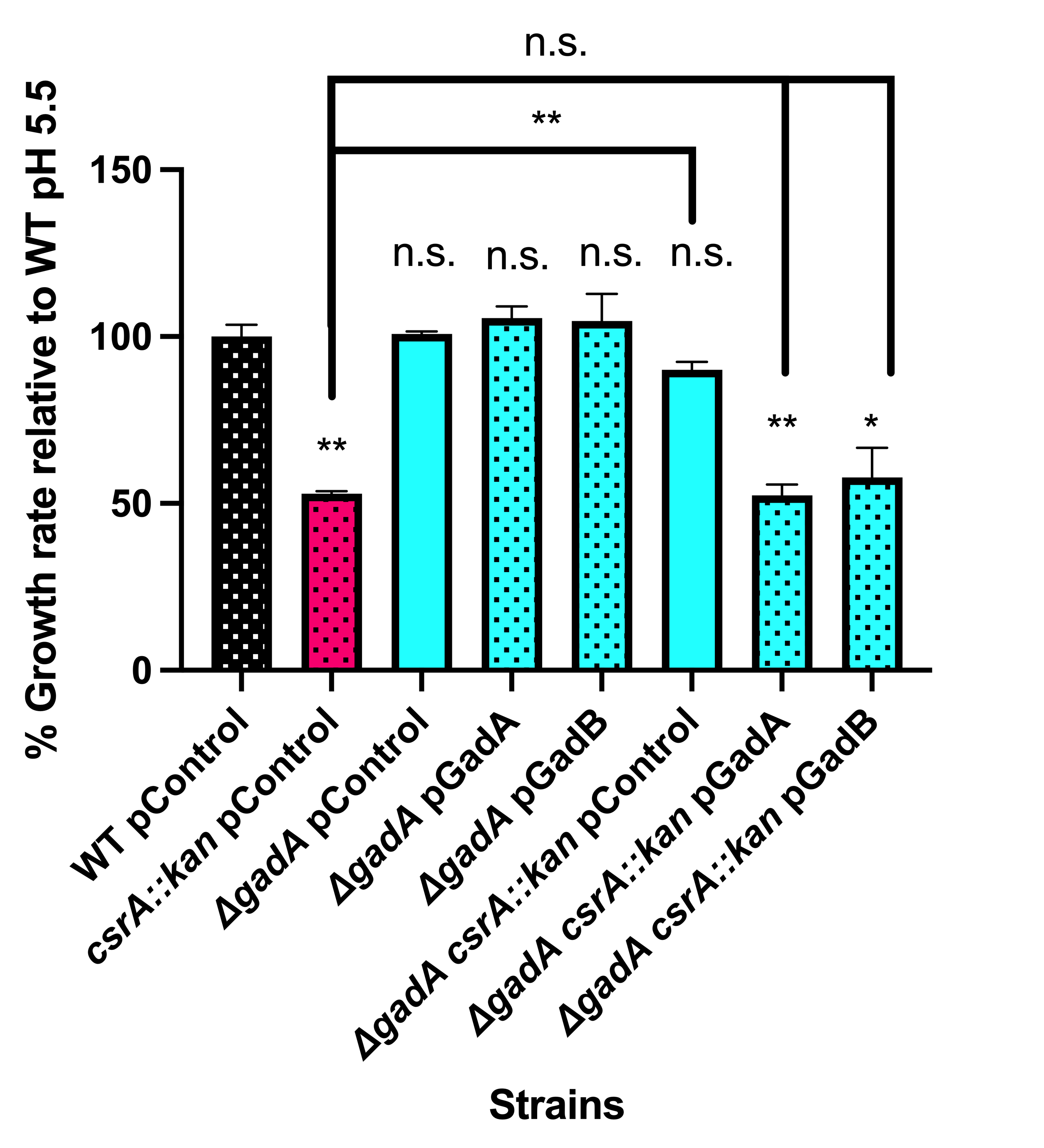


**FIG S4** Effect of *gadB* overexpression on growth of a Δ*gadA* strain. Growth rates (μ) of exponential phase *E. coli* strains in mildly acidic (pH 5.5) M9 media normalized to WT. Error bars represent standard deviation (sd) from at least three independent experiments. Statistical significance was determined using unpaired t tests and is denoted as follows: *, P<0.05; **, P<0.01; ***, P<.001. The absence of a comparison bar indicates a comparison to WT.


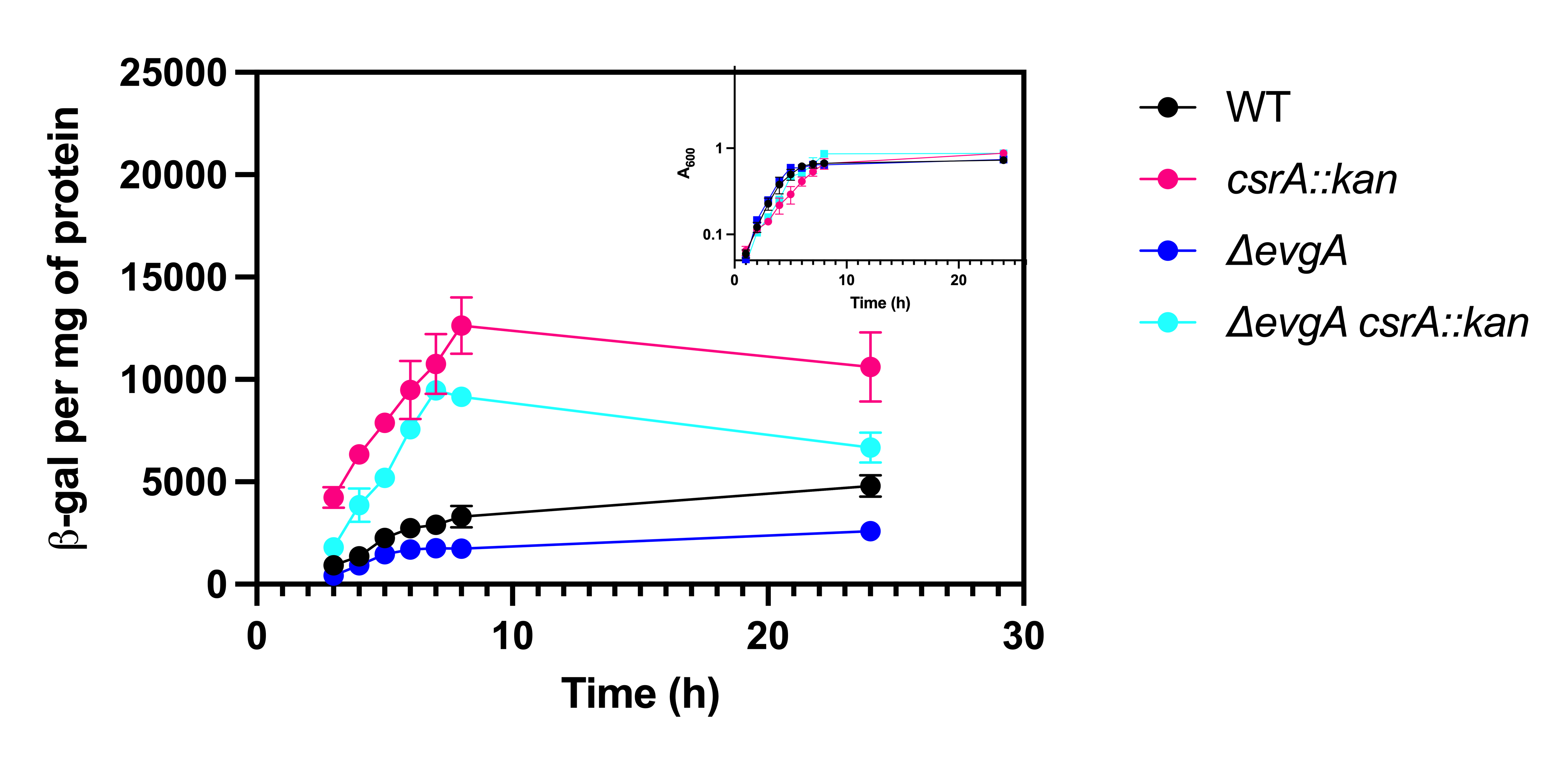


**FIG S5** Effects of *csrA* and *evgA* on expression of a *gadE’-‘lacZ* translational fusion. Growth is shown in the insert. Error bars represent standard deviation (sd) from three independent experiments.

**FIG S6** Effects of pH downshift on expression of *ydeP*, *csrA, csrB* and *csrC*. Effect of pH downshift on expression of (A) a *ydeP'-'lacZ* translational fusion, (B) a *csrA'-'lacZ* translational fusion, C) a *csrB-lacZ* transcriptional fusion, and D) a *csrC-lacZ* transcriptional fusion. Error bars represent standard deviation (sd) from three independent experiments. Statistical significance was determined using unpaired t tests and is denoted as follows: *, P<0.05; **, P<0.01.
